# Assessment and refinement of eukaryotic gene structure prediction with gene-structure-aware multiple protein sequence alignment

**DOI:** 10.1186/1471-2105-15-189

**Published:** 2014-06-14

**Authors:** Osamu Gotoh, Mariko Morita, David R Nelson

**Affiliations:** 1 Computational Biology Research Center (CBRC), National Institute of Advanced Industrial Science and Technology (AIST), Koto-ku, Tokyo 135-0064, Japan; 2 Department of Computational Biology, Graduate School of Frontier Sciences, The University of Tokyo, Kashiwa, Chiba 277-8561, Japan; 3 Department of Microbiology, Immunology and Biochemistry, University of Tennessee Health Science Center, Memphis, TN 38163, USA

**Keywords:** Genome annotation, Gene prediction, Gene structure, Multiple sequence alignment, Spliced alignment, Cytochrome P450, Ribosomal proteins

## Abstract

**Background:**

Accurate computational identification of eukaryotic gene organization is a long-standing problem. Despite the fundamental importance of precise annotation of genes encoded in newly sequenced genomes, the accuracy of predicted gene structures has not been critically evaluated, mostly due to the scarcity of proper assessment methods.

**Results:**

We present a gene-structure-aware multiple sequence alignment method for gene prediction using amino acid sequences translated from homologous genes from many genomes. The approach provides rich information concerning the reliability of each predicted gene structure. We have also devised an iterative method that attempts to improve the structures of suspiciously predicted genes based on a spliced alignment algorithm using consensus sequences or reliable homologs as templates. Application of our methods to cytochrome P450 and ribosomal proteins from 47 plant genomes indicated that 50 ~ 60 % of the annotated gene structures are likely to contain some defects. Whereas more than half of the defect-containing genes may be intrinsically broken, *i.e.* they are pseudogenes or gene fragments, located in unfinished sequencing areas, or corresponding to non-productive isoforms, the defects found in a majority of the remaining gene candidates can be remedied by our iterative refinement method.

**Conclusions:**

Refinement of eukaryotic gene structures mediated by gene-structure-aware multiple protein sequence alignment is a useful strategy to dramatically improve the overall prediction quality of a set of homologous genes. Our method will be applicable to various families of protein-coding genes if their domain structures are evolutionarily stable. It is also feasible to apply our method to gene families from all kingdoms of life, not just plants.

## Background

Recent progress in DNA sequencing technologies is making it possible to determine thousands of eukaryotic genomic sequences [1,2]. On an even larger scale, the China National Genebank has launched the Three Million Genomes (3 M) Project to sequence one million human genomes, one million plant and animal genomes and one million micro-ecosystem genomes [3]. To gain full use of the genomic information, the next step after sequencing is to annotate genes encoded in each genome. Several genome annotation pipelines have been established and actively used in a number of genome projects [4-6]. One of the most important products of genome annotation is a set of amino acid sequences translated from predicted protein-coding genes. Obviously, the quality of amino acid sequences, which in turn depends on the accuracy of gene prediction, profoundly affects the reliability of downstream analyses, such as functional implications, 3D-structure prediction of proteins, and evolutionary inference of the genes and species. The recently emerging method of co-evolutionary 3D structure prediction requires even larger numbers of accurate protein sequences [7-9] than the traditional template-based homology modelling methods [10].

Currently, three major categories of methods are used in computational prediction of protein-coding genes [11,12]. The *ab inito* methods rely on only the statistical features of the “query” genomic sequence to be analysed; the *de novo* or comparative genomic methods use the information of sequence conservation and variation between the query genome and one or more related “reference” genome(s); and the transcript-dependent methods use known transcript sequences, such as full-length cDNAs, ESTs, and proteins of cognate or related genomes as templates. The accuracy and coverage of gene prediction can be improved by combining several lines of information, and present annotation pipelines usually adopt several distinct categories of methods depending on the available information. Although human intervention can further improve the quality of annotation [13,14], manual annotation by experts is impractical to apply to all the genomes whose number is growing rapidly. Thus, the vast majority of genome annotations rely on high-throughput automated methods. However, assessment of the quality of the products of such procedures has not been well explored, mostly due to the scarcity of proper assessment methods.

In this work, we present gene-structure-aware multiple protein sequence alignment (GSA-MPSA) as a powerful tool to evaluate and refine a set of homologous (orthologous and paralogous) gene structures. A GSA-MPSA is constructed from translated amino-acid sequences supplemented with the location and phase of introns along the coding sequence (CDS) of the parental (predicted) genes. Our approach is based on the facts that (i) long insertions/deletions are rare among closely related homologous protein sequences and (ii) positions of introns in a set of homologous genes are generally well conserved [15]. Thus, we can expect high reliability of gene predictions if we observe few gaps and a concordant distribution of intron positions in the GSA-MPSA. Conversely, existence of long gaps or a discordant distribution of intron positions is a strong indicator of some defects including annotation errors.

Although many MPSA methods have been developed so far ( [16] for the latest reviews), none is designed to incorporate the information about exon-intron organization of the parental genes. Hence, we extended our iterative MPSA method Prrn [17,18] so that conserved intron positions should gain an additional bonus in the objective function to be optimized. The resultant MPSA labelled with intron positions is parsed to see the distribution of indels and also the distribution of the concordant/discordant intron sites with the help of an outlier statistical analysis. We have also extended our spliced alignment program Aln [19] so that it can use a generalized profile as the template. Generalized profiles have been used in internal routines of Prrn [20] to detect and evaluate gaps exactly and efficiently, where “generalized” means that various lengths of internal gaps, as well as ordinary residues, are treated in the form of a profile [21]. We have implemented a tool named “Refgs.pl” (refine gene structures) that tries to successively improve individual gene structures of the members constituting the GSA-MPSA by running Prrn and Aln in an iterative manner. To test the performance of Refgs.pl, we applied our method to the cytochrome P450 and ribosomal protein genes annotated in 47 plant genome datasets, most of which were obtained from Phytozome Ver. 9.0 [22]. These gene (super-) families were chosen as representatives of two distinct categories of genes, a divergent multi-gene superfamily that codes for enzymes and a set of unique gene families that code for individual subunits of a large protein-RNA complex, respectively. The results indicate that Refgs.pl is highly effective at detecting various kinds of defects including annotation errors, a majority of which can be corrected by the iterative refinement procedure. External tests with EST mapping and manual inspection indicated that nearly 99% of exon-intron boundaries are correctly assigned after a series of refinements by Refgs.pl, and the overall quality of gene prediction is comparable with that obtained by specialists in the field.

## Results

### Performance of spliced alignment with a profile template

**Figure 1:**
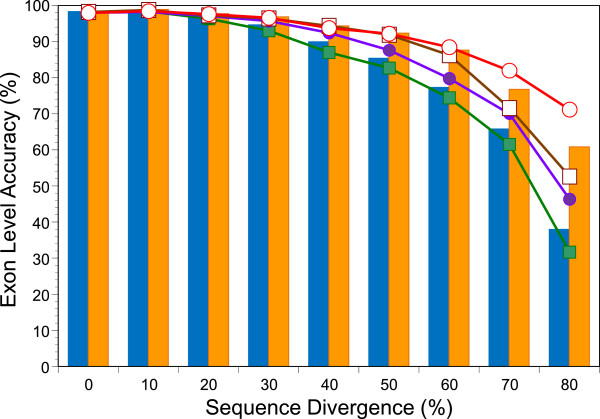
**Improved accuracy of spliced alignment with profile templates.** Alignment accuracy at the exon level (F-measure) with single amino-acid templates (blue bar) is compared with those using profile templates (orange bar) of the same sequence identity level. Specificities (circles) and sensitivities (squares) with single amino-acid templates (filled) and profile templates (open) are also shown by line graphs.

MSAs and profiles derived therefrom have been successfully used to improve the quality of various biological sequence analyses, such as remote sequence similarity search [23], prediction of secondary structure of proteins [24], protein fold recognition [25], and alignment itself [26]. However, no attempt appears to have been made to examine whether the use of a profile can improve the quality of a spliced alignment, although an appreciable number of spliced alignment programs have been developed so far [27] including GeneWise [28] that supports profile-HMM-based spliced alignment. To test the effects of profiles, we used the same dataset, P491, as that used in previous studies [29,30]. P491 consists of 491 human genes and their mouse orthologs together with a total of more than 71,000 homologous protein sequences used as the references (templates). In the original study, a sequence was randomly chosen from the reference set for each bin of sequence identity level. Here, we compare the results of such an examination with those obtained by a use of a profile derived from all sequences in each bin as the template. As shown in Figure 1, use of profiles significantly improves both sensitivity and specificity at the exon level. Figure 1 also indicates that prediction with a close reference, if one exists, may outperform that with a profile consisting of remote reference sequences. These observations afford a foundation for our template selection modes described in the next subsection.

### Assessment and refinement of gene structures by Refgs.pl

**Figure 2:**
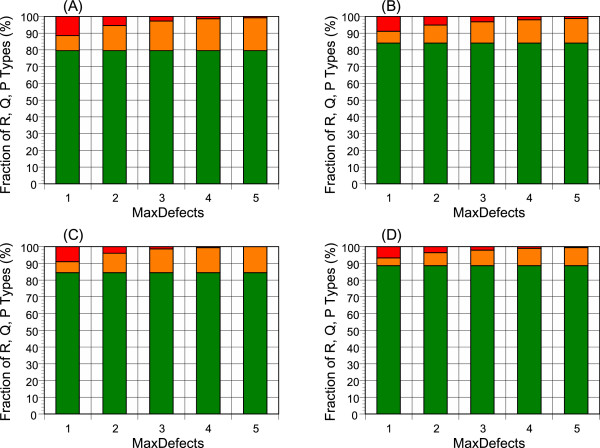
**Classification of predicted genes into three reliability types.** Each gene is classified into “R” (reliable, green), “Q” (questionable, orange), or “P” (pseudo, red) type according to the cumulated defect points. The “R” type has zero defect points, and *MaxDefect* value delimits the boundary between “Q” and “P” types. **(A)** and **(B)** show the results of P450s before and after CR-M1-PR iterative refinement, respectively, and **(C)** and **(D)** show the corresponding results for ribosomal proteins.

To examine how reliable predicted protein sequences produced by plant genome projects are, we first constructed GSA-MPSAs from individual clusters of P450 and ribosomal proteins as described in Methods. A cluster is an operational unit consisting of closely related orthologs and paralogs. By applying outlier analyses to the indels and local sequence variations, and also examining the distribution of intron positions within the GSA-MPSA, we categorized each predicted gene into “R” (reliable), “Q” (questionable), or “P” (pseudo) types. Figure 2 shows the fractions of these types as a function of the threshold value for the defect point between “Q” and “P” types (*MaxDefect*). In this and most other tests, the maximal cluster size (*MaxCluster*) is fixed to 50, approximately the number of genomes examined. The fraction of “R”-type sequences amounts to about 80% of all sequences tested. However, the percentage of the “R” sequences of the initial annotated genes is only about 55% as the clusters were constructed from the sequences that passed two preliminary criteria (Methods). Moreover, this simple evaluation method considerably overestimates “R” type genes, as the outlier analysis fails to detect abnormalities when the variance of the variables is large. For example, no indel outlier is detected in the N terminal part of the alignment shown in the Additional file 1: Figure S1A, although human eyes clearly perceive some abnormalities. Long indels that are not recognized as abnormal by the outlier analysis were actually prevailing among the GSA-MPSAs, some of which are exemplified in the Additional file 1: Figures S1A-F.

**Figure 3:**
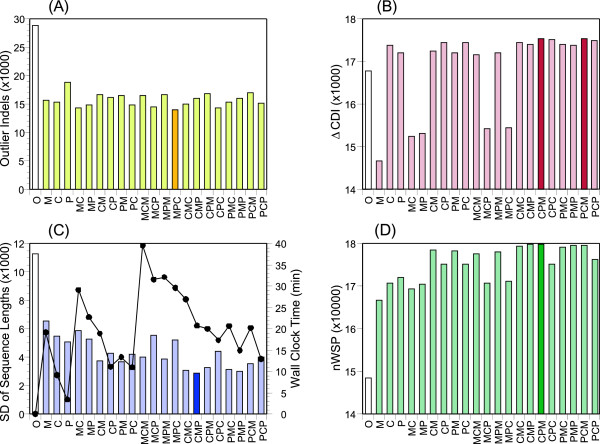
**Evaluation of GSA-MPSAs of P450 clusters viewed from various aspects. (A)** Total number of outlier indels. **(B)** Difference in the total numbers of concordant and discordant introns. **(C)** The sum of standard deviations of sequence lengths in variable regions. The total computational time spent for iterative refinement with 10 CPUs is also indicated. **(D)** The total sum of normalized weighted sum-of-pairs scores of all GSA-MPSAs. The results obtained from the original annotation are indicated by blank bars. Of the 21 combinations of template modes, the best combination for each aspect is indicated by the thick bar.

**Figure 4:**
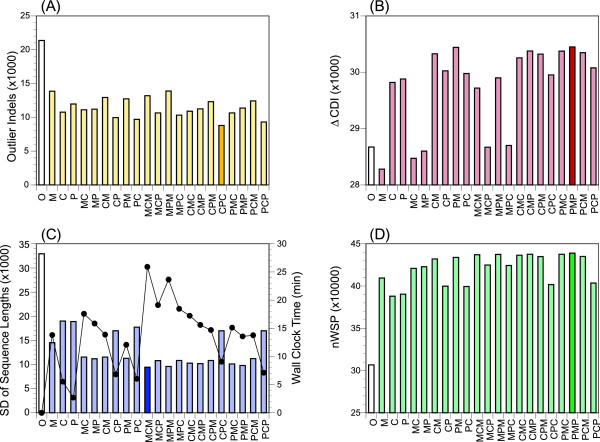
**Evaluation of GSA-MPSAs of ribosomal protein clusters viewed from various aspects. (A)** Total number of outlier indels. **(B)** Difference in the total numbers of concordant and discordant introns. **(C)** The sum of standard deviations of sequence lengths in the variable regions. The total computational time spent for iterative refinement with 10 CPUs is also indicated. **(D)** The total sum of normalized weighted sum-of-pairs scores of all GSA-MPSAs. The results obtained from the original annotation are indicated by blank bars. Of the 21 combinations of template modes, the best combination for each aspect is indicated by the thick bar.

It is naturally expected that the accuracy of prediction of individual gene structures would be increased by improving the quality of the GSA-MPSA in which the genes participate. We designed three template selection modes and their combinations to improve the quality of a GSA-MPSA by iteration (Methods). The quality of a GSA-MPSA was examined from various aspects, such as the number of outlier indels and local sequence variations, the numbers of concordant and discordant (lonesome) introns and their difference (*ΔCDI*), the standard deviation of sequence lengths in variable regions, and the normalized sum-of-pairs (*nSP*) or normalized weighted sum-of-pairs (*nWSP*) MSA score. Figures 3 and 4 show the results of the iterative refinement for P450 and ribosomal proteins, respectively, with respect to four representative features. These figures clearly indicate that the iterative procedures improve all the features, except for *ΔCDI* with the M1 mode (Methods) and the combination modes that initiate with the M1 mode. The M1 mode tends to overestimate exons when some member(s) in the cluster contain a foreign sequence(s) which acts as an erroneous template to incorporate false exons in other members, whereas the other two modes are robust in this respect. By contrast, the M1 mode is more effective than the other modes to fill in exonic regions that are missing in the original prediction. Thus proper combinations of M1 and the other two modes perform best; CR-M1-PR, PR-M1-PR, and CR-PR-M1 are generally the best combinations as judged from *nWSP* (or *nSP*) that incorporates various features into a single score. Wilcoxon signed rank tests indicated that the mutual merits of the three combinations were insignificant (*p* > 0.25 for P450 and *p* > 0.03 for ribosomal proteins).

**Figure 5:**
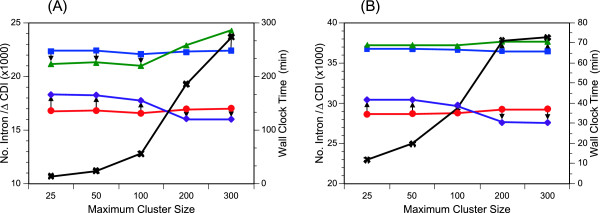
**Effects of MaxCluster on the ability for an iterative refinement to improve the number of concordant introns.** The *ΔCDI* values before (red filled circles) and after CR-M1-PR iteration (purple filled diamonds), together with the total number of introns before (blue filled squares) and after iteration (green filled triangles), are indicated as a function of *MaxCluster*. **(A)** P450s. **(B)** ribosomal proteins. The wall clock time spent for calculation with 10 CPUs in parallel is also shown by black crosses.

Figure 5 shows the effects of *MaxCluster* on the performance of iterative refinement. As most quantities used for evaluation mentioned above depend on a cluster size in complicated manners, we consider only *ΔCDI* that is not directly affected by a cluster size. For small *MaxClusters*, CR-M1-PR refinement clearly improves *ΔCDI* of both P450s and ribosomal proteins. However, for *MaxCluster* = 200 or 300, CR-M1-PR refinement worsens *ΔCD*, which appears to largely originate from over-prediction of extra exons/introns. The EST-based analyses described in the next subsection also indicate over-prediction of introns with *MaxCluster* = 200 or 300. Furthermore, the computational time increases near quadrically with *MaxCluster* (Figure 5). Thus, we may conclude that *MaxCluster* should be kept within a two-digit number, although the best choice would vary with the number of genomes under analysis, the relative frequencies of orthologs and paralogs, the qualities of initial annotation, and other factors.

### EST-based and manual assessments

Most genome annotation pipelines utilize the results of EST mapping on the relevant genome as a component of evidence-based prediction [31,32]. Hence, the false rates assessed through our mapping results are expected to be small. In fact, the sensitivity (*SN*) and specificity (*SP*) at the intron level (both ends should be correct) exceed 96% and 94%, respectively (Additional file 2: Table S1A, B), when all retrieved sequences before filtering were examined. The two steps of filtering improved both *SN* and *SP* by ~1% (Table S1C, D). Although marginal, CR-M1-PR refinement with *MaxCluster* = 25 or 50 further improved *SN* and *SP* (Table S1E, F) despite the fact that no information about EST mapping is incorporated in the refinement process.

**Figure 6:**
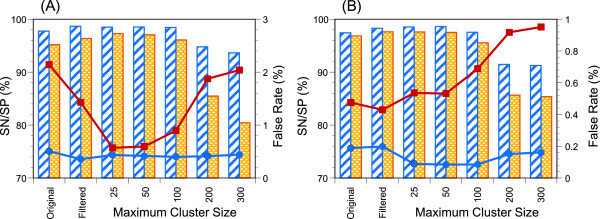
**EST-based assessment of gene prediction.** A true positive (TP) intron of a predicted gene is defined as that having the same genomic coordinates at the both ends as an EST-supported intron. A false positive (FP) intron is defined as a predicted intron whose genomic region is assigned to be exonic by the EST-mapping results. However, homology-supported true positive introns are counted not as FP but as TP in this analysis. A false negative (FN) intron is defined as an EST-supported intron whose genomic region is assigned to be exonic by the prediction. Sensitivity (blue hatched bar) is defined as 100 *TP/RI, where RI is the total number of EST-supported introns that overlap with the genomic regions predicted to be genic (exon or intron) by the prediction. Specificity (orange shaded bar) is defined as 100 *TP/QI, where QI is the total number of predicted introns that overlap with at least one EST-supported genic area. A false positive rate (red filled square) is defined as 100 *FP/QI, and a false negative rate (blue filled circle) is defined as 100 *FN/RI. **(A)** P450s. **(B)** ribosomal proteins.

In plants, the most prevalent alternative splicing events are the retained-intron type [33,34]. Hence, some EST sequences may represent unspliced or aberrantly spliced isoforms, which leads to an overestimate of false positive introns. To examine this possibility, we asked whether each false positive intron position might be conserved in other homologous gene(s). If the relevant intron is a lonesome intron, that intron is likely to be real false positive, but otherwise that intron can be regarded as a “homology-supported” true positive intron. Figure 6 shows the results after this correction (Table S1 represents both results before and after the correction). The most remarkable point observed in Figure 6 is that the fraction of “pure” false positive introns (lonesome introns whose corresponding regions are exonic according to the results of EST mapping) of P450s is dramatically reduced by the CR-M1-PR refinement, suggesting the efficacy of our refinement procedure. No improvement was observed for ribosomal proteins probably because the original false positive rate was already quite low.

**Figure 7:**
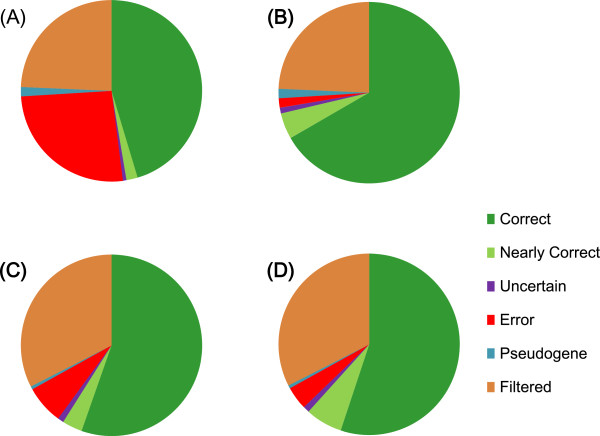
**Manual assessment of peach (A and B) and maize (C and D) P450 genes before (A and C) and after (B and D) CR-M1-PR refinement.** Near correct prediction implies that either the translational initiation site is ambiguous or the gene contains a frame shift or a premature termination codon within a predicted exon but otherwise looks intact. Uncertain prediction implies that the true gene organization cannot be predicted unambiguously, whereas pseudogene indicates that its gene organization is apparently singular. Filtered indicates the fraction of the genes in the original annotation that were filtered out by the two-step filtering.

One caveat of this type analysis is that the introns thus examined do not necessarily belong to the genes under investigation; if a prediction erroneously contains an extra genomic segment that is expressed as a distinct transcriptional unit, the EST-based assessment may regard such extra exons/introns as true positives. To synthetically evaluate the integrity of the predicted gene structures, we also conducted detailed analysis of the amino acid sequences of P450s encoded in two representative genomes, peach [35] and maize [36]. The peach gene models were obtained by a typical automated pipeline that combines several gene prediction algorithms, whereas ~60% of the maize P450 genes were manually curated by one of the present authors. The full maize genome was annotated by the Gramene pipeline [37], that takes advantage of extensive maize full length cDNAs and closely related well annotated genomes like rice. We found that GSA-MPSAs as shown in Additional file 1: Figure S1 are helpful for manual assessment as well, and could unambiguously infer true gene structures for more than 90 ~ 97% of seemingly genuine or nearly complete genes. Although almost all existing annotations disallow frame shifts and premature termination codons and hence the genes are truncated or the affected exons are skipped, our method properly accommodates such defects. If a gene harbours a single such defect but otherwise looks intact, we regard the gene as near complete, whereas if a plural number of defects are found, the gene is regarded as a pseudogene. The most common ambiguity is the location of a translational initiation site (TIS), when two or more initiation codons are closely arranged in the same reading frame. We regarded a variant as either erroneous or near correct depending on whether the N-terminal hydrophobic transmembrane domain, which is ubiquitous among all microsomal P450 proteins, is truncated or not. Besides this type of ambiguity, only three of peach and four of maize genes remained uncertain with respect to their exon-intron organizations. As summarized in Figure 7 and detailed in the Additional file 2: Table S2, the CR-M1-PR refinement augmented the number of correctly or near-correctly predicted P450 genes in the peach genome by 72 (24% of total), and only five genuine genes contained errors after the refinement. By contrast, CR-M1-PR refinement introduced 15 new errors that were absent in the initial annotation, while it remedied 25 errors in the original annotation. Thus, we may conclude that our refinement method is not perfectly accurate but at least as effective as manual curation by specialists in reducing the errors in gene prediction by an automated method.

## Discussion

For human and several model organisms [38-40], the catalogue of their protein-coding genes is nearly complete. The situation is completely different for the vast majority of organisms whose genomic sequences are recently revealed. Although high-throughput gene annotation pipelines are widely used, the accuracy of such products has not been well studied. To our knowledge, MisPred [41,42] is the sole practical tool to search for potentially erroneous annotations. To evaluate the reliability of a prediction, MisPred examines macroscopic features of the predicted amino acid sequences. Accordingly, MisPred is not necessarily effective to discover individual defects, especially subtle defects. Moreover, MisPred affords no way to correct annotation errors it finds. We have shown here that a GSA-MPSA constructed from close homologues provides rich information about the reliability of each predicted gene structure. Thanks to the recent progress in many genome projects undertaken in parallel, collection of appropriate homologs is now much easier than a few years ago. We have also shown that GSA-MPSA can facilitate not only evaluation but also refinement of the predicted gene structures. The improvement in the quality of prediction is demonstrated from various viewpoints as shown in Figures 3 and 4. However, a more direct impression would be obtained by looking into the alignments themselves. Additional file 1: Figure S1 presents several examples of GSA-MPSAs of the same genes before and after refinement. The smaller numbers of indels and better aligned intron positions after refinement compared with those in the original GSA-MPSAs will give an intuitive support for the efficacy of our refinement procedure.

In essence, our refinement strategy relies on the consensus or the “decision by majority” rule. In general, consensus-based approaches fail when the initial predictions are scarce, poor, or highly heterogeneous. In fact, of the 484 clusters of P450 and 1217 clusters of ribosomal proteins with *MaxCluster* = 50, the CR-M1-PR refinement worsened the *nWSP* scores for 20 and 43 clusters (211 and 368 sequences involved), whereas the *nWSP* scores remained unchanged for 38 and 119 clusters (164 and 580 sequences) and improved for the rest of the 426 and 1055 clusters (8083 and 13470 sequences), respectively. In addition, 126 P450 and 459 ribosomal protein sequences didn’t belong to any cluster due to the lower limit in the minimal size of clusters and had no chance to be evaluated or refined. Roughly speaking, therefore, our refinement procedure is effective or neutral for about 93 ~ 96% but inadequate for about 4 ~ 7% of sequences that passed the two filtering criteria (Methods). (Note that the fraction of inadequate cases for ribosomal proteins is inflated as the initial filtering imposed no limitation in length.) One potential way to cope with these difficult cases would be to improve the profile method so that more distant homologs can be used as the templates, or pre-annotated sequences from human curated datasets could be used. In addition, the 1000 plant transcriptome project now in progress [43] will provide a broad sampling of plant gene diversity including distant homologs currently lacking as templates.

By counting the defect points, we estimated that about 80 ~ 84% of the original sequences that passed the two filtering criteria are regarded as “R” type (Figure 1). By the CR-M1-PR refinement, the fraction of the “R” type genes are increased (Figure 1), and we estimate that about 58% of P450 and about 70% of ribosomal protein genes in the initial annotations are likely to be genuine. However, the CR-M1-PR refinement modified the gene structures of a large fraction of the original predictions. As a result, only about 40% of P450 and about 50% of ribosomal protein genes in the original annotations remain unmodified and are regarded as defect free after the refinement (Table 1). As demonstrated by manual assessments, not all the changes are improvements, but the above discussion about *nWSP* scores suggests that at least 90% of the modifications are likely to be real improvements.

**Table 1:** Numbers of sequences at various stages of data processing

**Gene**	**Retrieved**	**1**^**st **^**filter**	**2**^**nd **^**filter**	**Outside Clusters**	**Last stage R-type**	**Last stage Q-type**	**Un-modified**	**Un-modified R-type**
P450	12308	9016	8458	126	7111	905	5204	4935
(%)	(100)	(73.3)	(69.5)	(1.0)	(57.8)	(7.3)	(42.2)	(40.1)
RBP	18513	17930	14418	459	12953	966	9401	9196
(%)	(100)	(96.9)	(77.9)	(3.7)	(70.0)	(5.2)	(76.4)	(49.7)

As our method does not directly refer to the available cDNA/EST information, the assessment based on the EST mapping results can be regarded as an external test. We consider that the sensitivity and specificity of 97 ~ 98% at the intron level and 98 ~ 99% at the junction level are quite high. One reason for this high accuracy might be because the original annotations had already incorporated EST mapping information and some annotations had been manually curated. It is an interesting next theme to examine how our approach performs without relying on existing annotations.

In the present study, we analysed *ca*. 10^4^ P450 and ribosomal protein sequences. Although the Phytozome dataset are generally well organized, the formats of gene annotation of various resources are not completely uniform, rendering the preparation of the initial sets of amino acid sequences supplemented with parental gene structure information rather laborious. Once the initial setting was completed, however, the entire process including clustering, assessment, and refinement finished within an hour on our computer system (CentOS 6.2, Xeon E5-2687 W, 3.1GHz, 8 × 2 cores, 64GB memory, 2 TB hard disk) when *MaxCluster* is set to 50 or smaller (Figures 3 and 4). It was feasible because the post-clustering processes are executable in parallel, where we used ten cores/threads in the present experiments. Thus, it would not be much additional burden to analyse an order of magnitude larger number of sequences in a personal computational environment, and this may be scaled further with an institutional computer system.

## Conclusions

We have shown that GSA-MPSA-mediated refinement of eukaryotic gene structures is a useful automated strategy to dramatically improve the overall prediction quality of a set of homologous genes. Our method will be applicable to various families of protein-coding genes if their domain structures are evolutionarily stable. It is also feasible to apply our method to gene families in any kingdoms of life. The C++ source codes of Aln, Prrn, and Spaln, the engines of our strategy, are available from our web site [44]. The Perl scripts of Refgs.pl that organizes the refinement process together with source codes/scripts of the associated programs are also made available from the same site as Prrn/Aln.

## Methods

### Data retrieval and preparation of initial GSA-MPSA

**Figure 8:**
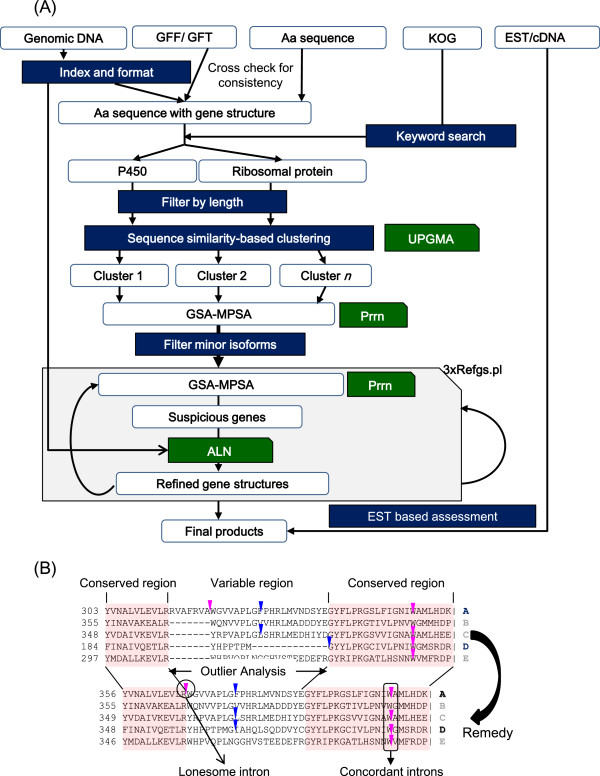
**The outline of the preparation and analyses of data. (A)** The workflow of data processing. **(B)** Conceptual demonstration of the Refgs.pl strategy. The phase 1 and phase 2 introns are indicated by magenta and blue triangles, respectively.

The outline of our method is illustrated in Figure 8. The genomic datasets we used are listed in the Additional file 2: Table S3. Note that the P450 genes of the species marked by an asterisk had been curated by one of the present authors. Each dataset consists of several files of which we used those containing genomic DNA sequences, predicted amino acid sequences, gene structures in the GFF or GFT format, functional annotation in the KOG format, and EST sequences. EST/cDNA sequences were also downloaded from UniGene [45], GenBank [46], and PlantGDB [47] databases if available. The genomic sequences were indexed and formatted to be used by Aln and also by Spaln [30,48] for fast transcript mapping. The amino acid sequences were retrieved by a keyword search against the KOG file, where we used either “P450” or “ribosomal protein” as the keyword, and amino acid sequences with the corresponding identifiers were extracted from the amino acid sequence file. If a KOG file is not present, the keywords were searched directly against the amino acid sequence file. Each amino acid sequence is supplemented with the information about the parental gene structure by referring to the corresponding entry in the GFF/GFT file. Consistency between a retrieved amino acid sequence and that translated from the genomic sequence was cross checked. Many minor discrepancies were found when the first and/or last codon is partial or the last coding exon is not followed by a termination codon, in which cases the first and/or last amino acid is modified to accord with the corresponding triplet in the genomic sequence. If the discrepancies were not trivial, the amino acid sequence was mapped on the relevant genome by Spaln, and the coordinates of the exon-intron boundaries were corrected according to the map results. We filtered out improper sequences by two criteria, *i.e.* the sequence length must be longer than a given threshold (400 for P450 and 0 for ribosomal proteins), and the number of ambiguous nucleotides (‘N’s) in the corresponding genomic sequence area must be smaller than a specific number (10 in the present study). The remaining amino acid sequences were compared with one another in the all-by-all fashion by a fast alignment-free method [49] to yield a distance matrix. The distance values were transformed into PAM scale (accepted point mutations per 100 sites) [50] by a polynomial regression. A UPGMA tree was constructed from the distance matrix, and the tree was divided into subtrees (clusters) by cutting the edges at a specific height (*MaxHeight*), and also by an upper limit on the membership (*MaxCluster*). A fixed value of *MaxHeight* = 120 is used throughout this study. With this threshold value and the infinitely large *MaxCluster*, each cluster roughly corresponds to a “family” of P450 proteins [51], *i.e.* mutual amino acid identities within each cluster should be about 40% or more. *MaxCluster* is introduced to restrict the maximal size of each cluster. We examined several values (25 ~ 300) for *MaxCluster*, whereas the lower limit of a cluster size (*MinCluster*) is fixed to three. This minimal cluster size eliminated 126 P450 and 459 ribosomal protein sequences from further analyses. Using each subtree as the guide tree, we constructed a GSA-MPSA as described in the next subsection in detail. At this stage, we applied the second filter to remove “minor” isoforms when several members within a cluster are derived from the same genomic region. The “major” isoform is defined as that having the highest average similarity with the cluster members other than the isoforms under question, whereas the minor isoforms are all the other isoforms. The MPSAs depleted of the minor isoforms were the starting material of our analyses. The numbers of P450 and ribosomal protein genes before and after the two steps of filtering are presented in Table 1.

### Methods for GSA-MPSA and spliced alignment

The original Prrn algorithm [17] attempts to maximize the weighted sum-of-pairs score by doubly-nested iterative refinement methods using the exact group-to-sequence or group-to-group pairwise alignment algorithm (Algorithm D in [52]) at each iterative step. To adapt Prrn to the present task, we have made several simplifications and extensions. First, a bonus is added to the objective function when the intron positions match with each other along the conceptual CDSs of an aligned pair of members. A match of intron positions means that not only the aligned columns but also the phases must be identical. Second, a double affine gap penalty can be used by option, while the forward algorithm is simplified compared with that of Prime [53], an extended version of Prrn that can handle long gaps more adequately than Prrn. Third, instead of the exact algorithm, an approximate algorithm (Algorithm C in [52]) is used at each step of pairwise alignment. With the Algorithm C, opening of gaps is evaluated exactly but the time-consuming optimization step is replaced by a more economical greedy method. Fourth, recalculation of pair weights was suppressed, i.e. the outer loop is run only once. Finally, Prrn now supports an “update” option with which the sequences with the same identifiers as newly given ones are first removed from the older MSA, new members are added one by one to the existing MSA, pair weights are calculated, and then iterative refinement is performed as usual. This option is particularly useful to keep consistency between the GSA-MPSA and the member sequences revised by new prediction of gene structures.

To align a genomic sequence and an MSA/GSA-MPSA, as well as a single amino acid sequence, we extended our spliced alignment program Aln [19] so that it can handle a generalized profile. Aln also adopts Algorithm C as the dynamic programming-based alignment engine when MSA/GSA-MPSA is assigned as the template. Matching intron positions are given a bonus as described above and earlier [30]. For most of the genomes we examined, species-specific parameter sets were available by the method described earlier [54]. If that was not the case, the parameter set of the evolutionarily closest species was used. Aln shares many subroutines with Spaln in common, and is essentially the same as Spaln with the –Q0 option when a single protein sequence is given as the template. However, unlike Spaln, Aln does not currently support the anchoring-based faster calculation mode.

### Assessment of predicted gene structures with GSA-MPSA

To find unusually long insertions or deletions in the given GSA-MPSA, we first locate conserved blocks within the GSA-MPSA by a heuristic score-based method [55] (Figure 8B). The lengths (numbers of residues) of the individual members within each variable region flanked by or outside such conserved blocks are subjected to recursive outlier analyses with Dixon’s method [56]. The significance level α is adjustable but α = 0.1 is used throughout this study. Long deletions at either end are specifically marked to indicate that the target genomic region should be extended more than usual in the next prediction cycle. Within each variable region, average sequence divergence between one member and the rest of the members is calculated in turn, and the set of divergence values are also subjected to the outlier analyses. This test is incorporated primarily to detect compensating frame shifts and also to detect alternatively spliced exons. At each time of outlier detection in either of the tests, a “defect point” of one is added to the corresponding member. The defect point is also incremented by one if the host gene has at least one “lonesome” or “discordant” intron, where a lonesome intron is an intron that finds no mate at the same position in other members (Figure 8B). The predicted genes were further examined for frame shift(s), premature termination codon(s), or ambiguous residue(s). Every such abnormality contributes to the defect point total by two. If the total defect point is zero, we assign the label “reliable” or “R”, if greater than zero and less than a given threshold, *MaxDefect*, we assign “questionable” or “Q”, and otherwise we assign “Pseudogene” or “P” to that member. We used *MaxDefect* = 2 in this study unless specifically remarked. In addition, we calculated several quantities, including the total number of outliers, total number of lonesome introns, variance in sequence lengths within the variable regions, and normalized sum-of-pairs (*nSP*) or normalized weighted sum-of-pairs (*nWSP*) score of the alignments. The normalization is taken with respect to both the number of sequence pairs in an MPSA and the length (number of columns) of the MPSA.

In addition to the GSA-MPSA-based assessment mentioned above, we also tried two other approaches. One is an automated method based on available EST/cDNA sequences; the EST/cDNA sequences were mapped on the cognate genomic sequence by Spaln [48] with the –LS option for local similarity search, and exon/intron boundaries thus inferred were compared with those of predicted genes. The other approach is manual inspection of various data, *e.g.* intactness of important linear motifs conserved among nearly all P450 protein sequences [57,58]. Because of the intrinsically subjective nature and the high human cost, we experimentally applied this approach to only P450 genes in two representative genomes (peach and maize).

### Iterative refinement of gene structures by Refgs.pl

Refgs.pl is a Perl script that achieves a cycle of assessments by Prrn and alignments by Aln until predicted gene structures no longer change, no “Q” gene remains, or up to a pre-specified number of times. We designed three modes which differ from one another by the choice of the templates. The “minus one” (M1) mode withdraws one sequence from the existing GSA-MPSA, and the structure of the corresponding gene is re-examined using the profile constructed from all the rest of the members. The GSA-MPSA is updated with the amino acid sequence translated from the revised gene, and the process is repeated in turn for every member. The “closest reliable” (CR) and the “reliable profile” (PR) modes update only the genes that are assigned “Q”; the former selects the template that is most similar to the gene in question from the “R” category, whereas the latter uses the profile derived from all the sequences in the “R” category as the template. We also examined all possible combinations of the three modes up to three series so that no consecutive modes should be identical, i.e. M1, CR, PR, M1-CR, …, PR-CR, M1-CR-M1, …, PR-CR-PR.

## Abbreviations

CDS: Coding sequence; EST: Expressed sequence tag; GSA-MPSA: Gene-structure-aware multiple protein sequence alignment; nSP: Normalized sum-of-pairs score; nWSP: Normalized weighted sum-of-pairs score.

## Competing interests

The authors declare that they have no competing interests.

## Authors’ contributions

OG conceived this study, drafted the manuscript, wrote most of the computer programs described herein, and performed a part of data analyses. MM wrote a fraction of computer programs and performed a part of data analyses. DRN participated in the design of this study, performed manual assessment of P450 gene annotations, and participated in improving the draft. All authors read and approved the final manuscript.

## Supplementary Material

Additional file 1: Figure S1 Examples of GSA-MPSA before and after iterative refinement. Intron positions are indicated by triangles: phase 0 (orange), phase 1 (magenta), and phase 2 (blue). A, C, and E show the results from P450 clusters 1, 4, and 8, respectively, and B, D, and F show the results from ribosomal protein clusters 1, 4, and 1098, respectively, with *MaxCluster* = 50. For each cluster, CR-M1-PR refinement was performed. Click here for file

Additional file 2: Table S1 EST-based assessment of gene prediction. NoEST: number of ESTs that overlap with the genic regions of P450 (A, C, E) or ribosomal protein (B, D, F) genes before (A, B) or after (C, D) two-step filtering, and after CR-M1-PR refinement (E, F). NoIntR: total number of introns supported by the ESTs that overlap with the predicted genic regions. NoIntQ: total number of introns in the predicted genes that overlap with at least one of EST mapped areas. IntTP: number of predicted introns that have the same genomic coordinates at the both ends as at least one EST-supported intron. IntFP: number of predicted introns whose genomic regions are assigned as exonic by the EST mapping. IntFN: number of EST-supported introns whose genomic regions are assigned as exonic by prediction. JuncTP: number of predicted exon-intron junctions that have the same genomic coordinates as at least one EST-supported intron. IntHS_TP: number of homology-supported true positive introns. For raw data, IntSn = 100xIntTP/NoIntR, IntSp = 100xIntTP/NoIntQ, JncSn = 50xJncTP/NoIntR, JncSp = 50xJncTP/NoIntQ, FPR = 100xIntFP/NoIntQ, and FNR = 100xIntFN/NoIntR. After correction for homology-supported introns, IntTP, JncTP, and IntFP are replaced by (IntTP + IntHS_TP), (JncTP + 2xIntHS_TP), and (IntFP-IntHS_TP), respectively. **Table S2:** Manual assessment of peach (A) and maize P450 (B) P450 genes. Seemingly correct and nearly correct predictions are indicated by the letter “C” and “c”, respectively. Near correct prediction implies that either the translational initiation site is ambiguous or the gene contains a frame shift or a premature termination codon within a predicted exon but otherwise looks intact. The letters “E”, “U”, “P”, and “F” respectively indicate erroneous prediction, uncertain prediction, apparent pseudogene, and gene fragment, respectively. **Table S3:** List of resources of sequences and annotations. An asterisk indicates that P450 genes of that species were once manually curated by one of the authors. Click here for file
