# Supplementary material for: Assessment and refinement of eukaryotic gene structure prediction with gene-structure-aware multiple protein sequence alignment
Source: BMC Bioinformatics. 2014 Jun 14;15:189. doi: 10.1186/1471-2105-15-189 (PMC4065584; doi:10.1186/1471-2105-15-189)

(before)

Fig. S1A: An example of GSA-MPSA

▼phase0 ▼phase1 ▼phase2

(after)

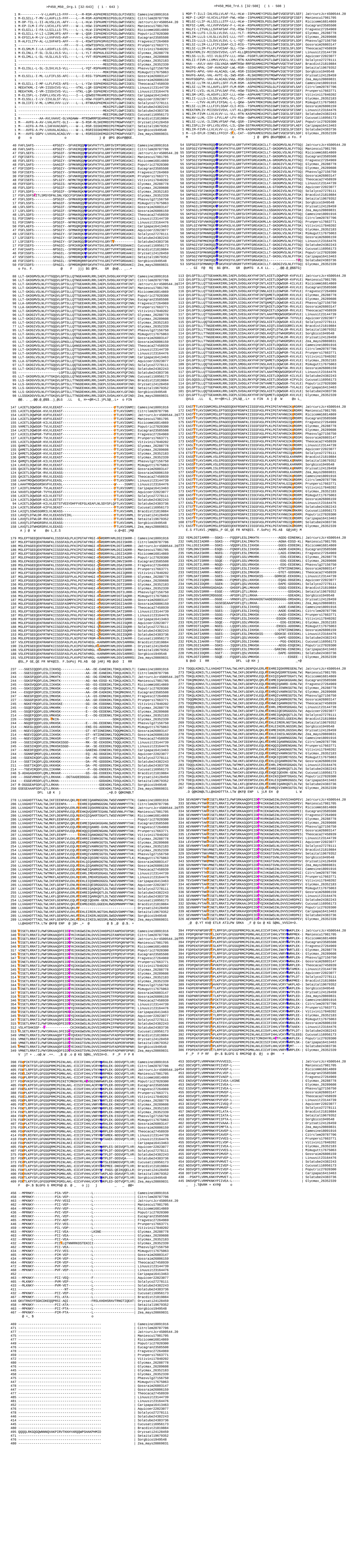

Fig. S1B: An example of GSA-MPSA

phase0 phase1 phase2

(before)

```
>RBP_M50_Org.1 [15:368]  ( 1 - 368 )

  1 M-----------------------------------------------------------| Aquicoer22023107
  1 M-----------------------------------------------------------| Arablyra16051447
  1 M-----------------------------------------------------------| Arabthal19639586
  1 M-----------------------------------------------------------| Arabthal19643610
  1 ------------------------------------------------------------| Ricicomm16828883
  1 MR----------------------------------------------------------| Caripapa16415391
  1 M-----------------------------------------------------------| Oryzsati24147382
  1 M-----------------------------------------------------------| Zea_mays20838612
  1 M-----------------------------------------------------------| Zea_mays20839405
  1 M-----------------------------------------------------------| Zea_mays20865706
  1 M-----------------------------------------------------------| Zea_mays20841553
  1 M-----------------------------------------------------------| Setaital19693586
  1 MW----------------------------------------------------------| Cucusati16966640
  1 M-----------------------------------------------------------| Cucusati16971357
  1 MPLPKAKQEETYGGPRSPLGRRQKSLPLKTEGTSDYSKPKRTRRGIGSPFTLFLGRNSGN| Gossraim26824973
    @

  2 ------------------------------------------------------------| Aquicoer22023107
  2 ------------------------------------------------------------| Arablyra16051447
  2 ------------------------------------------------------------| Arabthal19639586
  2 ------------------------------------------------------------| Arabthal19643610
  1 ------------------------------------------------------------| Ricicomm16828883
  3 ----------------------------------------------------------NI| Caripapa16415391
  2 ------------------------------------------------------------| Oryzsati24147382
  2 ------------------------------------------------------------| Zea_mays20838612
  2 ------------------------------------------------------------| Zea_mays20839405
  2 ------------------------------------------------------------| Zea_mays20865706
  2 ------------------------------------------------------------| Zea_mays20841553
  2 ------------------------------------------------------------| Setaital19693586
  3 ------------------------------------------------------------| Cucusati16966640
  2 ------------------------------------------------------------| Cucusati16971357
 61 GNSKPLSGWLNGFGRILNKRVCEIPVPLFFAARPFNQQISDVMKQLSKKAASNCSEILS-| Gossraim26824973

  2 --------------------GGLDGEQKQLINKLVNFRMKEGKRTRVRAIVYQTFHRPAR| Aquicoer22023107
  2 --------------------GGLDGEQKLLIKKLVNFRMKEGKRTRVRAIVYQTFHRPAR| Arablyra16051447
  2 --------------------GGLDGEQKLLIKKLVNFRMKEGKRTRVRAIVYQTFHRPAR| Arabthal19639586
  2 --------------------GGLDGEQKLLIKKLVNFRMKEGKRTRVRAIVYQTFHRPAR| Arabthal19643610
  1 --------------------------------------MKEGKRTRVRAIVYQTFHRLAR| Ricicomm16828883
  5 NISASSSKAASNCSEILSYMGGLDGEQKQLIKKLVNFRMKEGKRTRVRAIVYQTFHRPAR| Caripapa16415391
  2 --------------------GDFDGEQKELIKKLVNFRMIDGKRTRVRAIVYKTFHRLAR| Oryzsati24147382
  2 --------------------GDFDGEQKELIKKLVNFRMIDGKRTRVRAIVYKTFHRLAR| Zea_mays20838612
  2 --------------------GDFDGEQKELIKKLVNFRMIDGKRTRVRAIVYKTFHRLAR| Zea_mays20839405
  2 --------------------GDFDGEQKELIKKLVNFRMIDGKRTRVRAIVYKTFHRLAR| Zea_mays20865706
  2 --------------------GDFDGEQKELIKKLVNFRMIDGKRTRVRAIVYKTFHRLAR| Zea_mays20841553
  2 --------------------GDFDGEQKELINKLVNFRMIDGKRTRVRAIVYKTFHRLAR| Setaital19693586
  3 --------------------GGLDGEQKELIKKLVNFRMKEGKRTKVRVILSQTLNRPAQ| Cucusati16966640
  2 --------------------GGLDGEQKELIKKLVNFRMKEGKRTRVRAILYQTLNRPAQ| Cucusati16971357
120 ------------------YMGGLDGEQKQLIKKLVNFRMKE-------------------| Gossraim26824973
                    . o_.__+ @@j+@@_$+M _.++.+@+ @@ j.oj+ .j

 42 -TERDVIKLMVDAVEHIKPICEVEKVGVAGKIYDVPGIVARDRQQTLAIRWILGAAFKRR| Aquicoer22023107
 42 -TERDVIKLMVDAVENIKPICEVAKVGVAGTIYDVPGIVARDRQQTLAIRWILEAAFKRR| Arablyra16051447
 42 -TERDVIKLMVDAVENIKPICEVAKVGVAGTIYDVPGIVARDRQQTLAIRWILEAAFKRR| Arabthal19639586
 42 -TERDVIKLMVDAVENIKPICEVAKVGVAGTIYDVPGIVARDRQQTLAIRWILEAAFKRR| Arabthal19643610
 23 -TERDVIKLMVDAIDNIKPICEVEKVGVAGTIYDVPGIVARDRQQTLAIRWILEAAFKRR| Ricicomm16828883
 65 RTERDVIQFLVDAVENIKPICEVEKVRVAGTIYDVPGIVARDRQQTLAIRWILEAAFKRR| Caripapa16415391
 42 -TERDVIKLMVDAVDNIKPICEVVKVGVAGTIYDVPGILARDRQQTLAIRWILGAAFKRR| Oryzsati24147382
 42 -TERDVIKLMVDAVDNIKPICEVVKVGVAGTIYDVPGIVARDRQQTLAIRWILGAAFKRR| Zea_mays20838612
 42 -TERDVIKLMVDAVDNIKPICEVVKVGVAGTIYDVPGIVARDRQQTLAIRWILGAAFKRR| Zea_mays20839405
 42 -TERDVIKLMVDAVDNIKPICEVVKVGVAGTIYDVPGIVARDRQQTLAIRWILGAAFKRR| Zea_mays20865706
 42 -TERDVIKLMVDAVDNIKPICEVVKVGVAGTIYDVPGIVARDRQQTLAIRWILGAAFKRR| Zea_mays20841553
 42 -TERDVIKLMVDAVDNIKPICEVVKVGVAGTIYDVPGIVARDRQQTLAIRWILGAAFKRR| Setaital19693586
 43 -TERDGIKLMVEAVENIKPICEVEKVRVATTFYGVPGIVANDRQQTLAIRWIFEAAFKRR| Cucusati16966640
 42 -TERDGIKLMVEAVENIKPICEVEKVRVAATFYDVPGIVARDRQQTLAIRWILEAAFKRR| Cucusati16971357
143 --------------ENIKPICEVEKVRIAGTIYDVPGIVAKDRQQTLAIRWILEAAFKRR| Gossraim26824973
     ._+_ @jo@@_.@_jIKPICEV KV @A. oY VPGI@AjDRQQTLAIRWIo AAFKRR

101 ISYRISLEKCSFAEILDAYRKRGIARKKRENLHGLASTNR--RFAHFR------------| Aquicoer22023107
101 ISYRISLEKCSFAEILDAYQKRGSARRKRENLHGLASTNR--SFAHFR------------| Arablyra16051447
101 ISYRISLEKCSFAEILDAYQKRGSARRKRENLHGLASTNR--SFAHFR------------| Arabthal19639586
101 ISYRISLEKCSFAEILDAYQKRGSARRKRENLHGLASTNR--SFAHFR------------| Arabthal19643610
 82 ISYRISLEKCSFAEILDAYRKRGIA-----------------------------------| Ricicomm16828883
125 ISYRISLEKCSFDEILDAYKKMGIARKKRENLHGLASTNR--SFAHFR------------| Caripapa16415391
101 ISYRISLEKCSFAEILDAYRKRGISRKRRGNLHGLASTNR--SFAHFR------------| Oryzsati24147382
101 ISYRISLEKCSFAEILDAYRKRGISRKRRENLHGLASTNR--SFAHFR------------| Zea_mays20838612
101 ISYRISLEKCSFAEILDAYRKRGISRKRRENLHGLASTNR--SFAHFR------------| Zea_mays20839405
101 ISYRISLEKCSFAEILDAYRKRGISRKRRENLHGLASTNR--SFAHFR------------| Zea_mays20865706
101 ISYRISLEKCSFAEILDAYRKRGISRKRRENLHGLASTNR--SFAHFR------------| Zea_mays20841553
101 ISYRISLEKCSFAEILDAYRKRGISRKRRKNLHGLASTNR--SFAHFR------------| Setaital19693586
102 ISYKISLEKCSFDEILDAYRKRGIARKKRENLHRLASTNR--SFAHFR------------| Cucusati16966640
101 ISYRISLEKCSFDEILDAYRKRGIARKKRENLHRLASTNR--SFAHFR------------| Cucusati16971357
189 ISYRISLEKCSFAEILDAYRKRGIARKKRENLHGLSSTNR[illegible]GPFTPFRLTRGADLINALQ| Gossraim26824973
    ISY+ISLEKCSF EILDAYjK G .++++ _@+ @..._+   $. $+

147 ------------------------------------------------WW----------| Aquicoer22023107
147 ------------------------------------------------WW----------| Arablyra16051447
147 ------------------------------------------------WW----------| Arabthal19639586
147 ------------------------------------------------WW----------| Arabthal19643610
107 ------------------------------------------------------------| Ricicomm16828883
171 ------------------------------------------------WW----------| Caripapa16415391
147 ------------------------------------------------WW----------| Oryzsati24147382
147 ------------------------------------------------WW----------| Zea_mays20838612
147 ------------------------------------------------WW----------| Zea_mays20839405
147 ------------------------------------------------WW----------| Zea_mays20865706
147 ------------------------------------------------WW----------| Zea_mays20841553
147 ------------------------------------------------WW----------| Setaital19693586
148 ------------------------------------------------WW----------| Cucusati16966640
147 ------------------------------------------------WW----------| Cucusati16971357
249 REGSQVSHVVNFWGQAKVRARHLAMNSVTGSLSLCLVLASQVGIDSSERWACHVKAGPSN| Gossraim26824973
                                                     $

149 --------                                                    | Aquicoer22023107
149 --------                                                    | Arablyra16051447
149 --------                                                    | Arabthal19639586
149 --------                                                    | Arabthal19643610
107 --------                                                    | Ricicomm16828883
173 --------                                                    | Caripapa16415391
149 --------                                                    | Oryzsati24147382
149 --------                                                    | Zea_mays20838612
149 --------                                                    | Zea_mays20839405
149 --------                                                    | Zea_mays20865706
149 --------                                                    | Zea_mays20841553
149 --------                                                    | Setaital19693586
150 --------                                                    | Cucusati16966640
149 --------                                                    | Cucusati16971357
309 DCTKSLRH                                                    | Gossraim26824973
```

(after)

```
>RBP_M50_Trd.1 [15:150]  ( 1 - 150 )

  1 M-GGLDGEQKQLINKLVNFRMKEGKRTRVRAIVYQTFHRPAR-TERDVIKLMVDAVEHIK| Aquicoer22023107
  1 M-GGLDGEQKLLIKKLVNFRMKEGKRTRVRAIVYQTFHRPAR-TERDVIKLMVDAVENIK| Arablyra16051447
  1 M-GGLDGEQKLLIKKLVNFRMKEGKRTRVRAIVYQTFHRPAR-TERDVIKLMVDAVENIK| Arabthal19639586
  1 M-GGLDGEQKLLIKKLVNFRMKEGKRTRVRAIVYQTFHRPAR-TERDVIKLMVDAVENIK| Arabthal19643610
  1 M-GDFDGEQKELIKKLVNFRMIDGKRTRVRAIVYKTFHRLAR-TERDVIKLMVDAVDNIK| Oryzsati24147382
  1 M-GDFDGEQKELIKKLVNFRMIDGKRTRVRAIVYKTFHRLAR-TERDVIKLMVDAVDNIK| Zea_mays20838612
  1 M-GDFDGEQKELIKKLVNFRMIDGKRTRVRAIVYKTFHRLAR-TERDVIKLMVDAVDNIK| Zea_mays20839405
  1 M-GDFDGEQKELIKKLVNFRMIDGKRTRVRAIVYKTFHRLAR-TERDVIKLMVDAVDNIK| Zea_mays20865706
  1 M-GDFDGEQKELIKKLVNFRMIDGKRTRVRAIVYKTFHRLAR-TERDVIKLMVDAVDNIK| Zea_mays20841553
  1 M-GDFDGEQKELINKLVNFRMIDGKRTRVRAIVYKTFHRLAR-TERDVIKLMVDAVDNIK| Setaital19693586
  1 MWGGLDGEQKELIKKLVNFRMKEGKRTKVRVILSQTLNRPAQ-TERDGIKLMVEAVENIK| Cucusati16966640
  1 M-GGLDGEQKELIKKLVNFRMKEGKRTRVRAILYQTLNRPAQ-TERDGIKLMVEAVENIK| Cucusati16971357
  1 M-GGLDGEQKQLIKKLVNFRMKEGKRTRVRAIVYQTFHRLAR-TERDVIKLMVDAIDNIK| Ricicomm16828883
  1 M-GGLDGEQKQLIKKLVNFRMKEGKETRVRAIFYQTFHRPAR-TERDVIKLMVDAVENIK| Gossraim26824973
  1 M-GGLDGEQKQLIKKLVNFRMKEGKRTRVRAIVYQTFHRPARRTERDVIQFLVDAVENIK| Caripapa16415391
    M G oDGEQK LIjKLVNFRM _GKjT+VR Io jTojR Aj TERD Ijo@V_A@_jIK

 59 PICEVEKVGVAGKIYDVPGIVARDRQQTLAIRWILGAAFKRRISYRISLEKCSFAEILDA| Aquicoer22023107
 59 PICEVAKVGVAGTIYDVPGIVARDRQQTLAIRWILEAAFKRRISYRISLEKCSFAEILDA| Arablyra16051447
 59 PICEVAKVGVAGTIYDVPGIVARDRQQTLAIRWILEAAFKRRISYRISLEKCSFAEILDA| Arabthal19639586
 59 PICEVAKVGVAGTIYDVPGIVARDRQQTLAIRWILEAAFKRRISYRISLEKCSFAEILDA| Arabthal19643610
 59 PICEVVKVGVAGTIYDVPGILARDRQQTLAIRWILGAAFKRRISYRISLEKCSFAEILDA| Oryzsati24147382
 59 PICEVVKVGVAGTIYDVPGIVARDRQQTLAIRWILGAAFKRRISYRISLEKCSFAEILDA| Zea_mays20838612
 59 PICEVVKVGVAGTIYDVPGIVARDRQQTLAIRWILGAAFKRRISYRISLEKCSFAEILDA| Zea_mays20839405
 59 PICEVVKVGVAGTIYDVPGIVARDRQQTLAIRWILGAAFKRRISYRISLEKCSFAEILDA| Zea_mays20865706
 59 PICEVVKVGVAGTIYDVPGIVARDRQQTLAIRWILGAAFKRRISYRISLEKCSFAEILDA| Zea_mays20841553
 59 PICEVVKVGVAGTIYDVPGIVARDRQQTLAIRWILGAAFKRRISYRISLEKCSFAEILDA| Setaital19693586
 60 PICEVEKVRVATTFYGVPGIVANDRQQTLAIRWIFEAAFKRRISYKISLEKCSFDEILDA| Cucusati16966640
 59 PICEVEKVRVAATFYDVPGIVARDRQQTLAIRWILEAAFKRRISYRISLEKCSFDEILDA| Cucusati16971357
 59 PICEVEKVGVAGTIYDVPGIVARDRQQTLAIRWILEAAFKRRISYRISLEKCSFAEILDA| Ricicomm16828883
 59 PICEVEKVRIAGTIYDVPGIVAKDRQQTLAIRWILEAAFKRRISYRISLEKCSFAEILDA| Gossraim26824973
 60 PICEVEKVRVAGTIYDVPGIVARDRQQTLAIRWILEAAFKRRISYRISLEKCSFDEILDA| Caripapa16415391
    PICEV KV @A. oY VPGI@AjDRQQTLAIRWIo AAFKRRISY+ISLEKCSF EILDA

119 YRKRGIARKKRENLHGLASTNRRFAHFRWW                              | Aquicoer22023107
119 YQKRGSARRKRENLHGLASTNRSFAHFRWW                              | Arablyra16051447
119 YQKRGSARRKRENLHGLASTNRSFAHFRWW                              | Arabthal19639586
119 YQKRGSARRKRENLHGLASTNRSFAHFRWW                              | Arabthal19643610
119 YRKRGISRKRRGNLHGLASTNRSFAHFRWW                              | Oryzsati24147382
119 YRKRGISRKRRENLHGLASTNRSFAHFRWW                              | Zea_mays20838612
119 YRKRGISRKRRENLHGLASTNRSFAHFRWW                              | Zea_mays20839405
119 YRKRGISRKRRENLHGLASTNRSFAHFRWW                              | Zea_mays20865706
119 YRKRGISRKRRENLHGLASTNRSFAHFRWW                              | Zea_mays20841553
119 YRKRGISRKRRKNLHGLASTNRSFAHFRWW                              | Setaital19693586
120 YRKRGIARKKRENLHRLASTNRSFAHFRWW                              | Cucusati16966640
119 YRKRGIARKKRENLHRLASTNRSFAHFRWW                              | Cucusati16971357
119 YRKRGIARKKRENLHGLASTNRSFAHFRWW                              | Ricicomm16828883
119 YRKRGIARKKRENLHGLSSTNRSFAHFRWW                              | Gossraim26824973
120 YKKMGIARKKRENLHGLASTNRSFAHFRWW                              | Caripapa16415391
    YjK G .R++R NLH L.STNR FAHFRWW
```

Fig. S1C: An example of GSA-MPSA

▼phase0 ▼phase1 ▼phase2

(before)

(after)

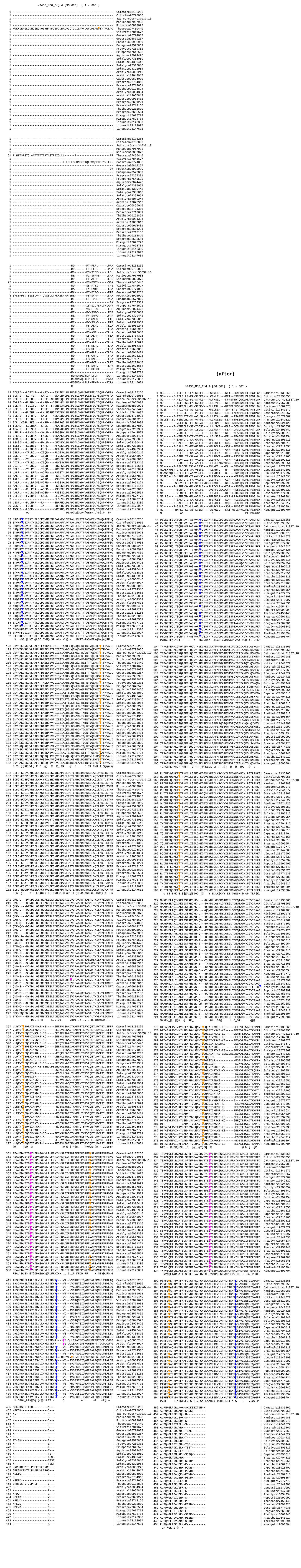

Fig. S1D: An example of GSA-MPSA

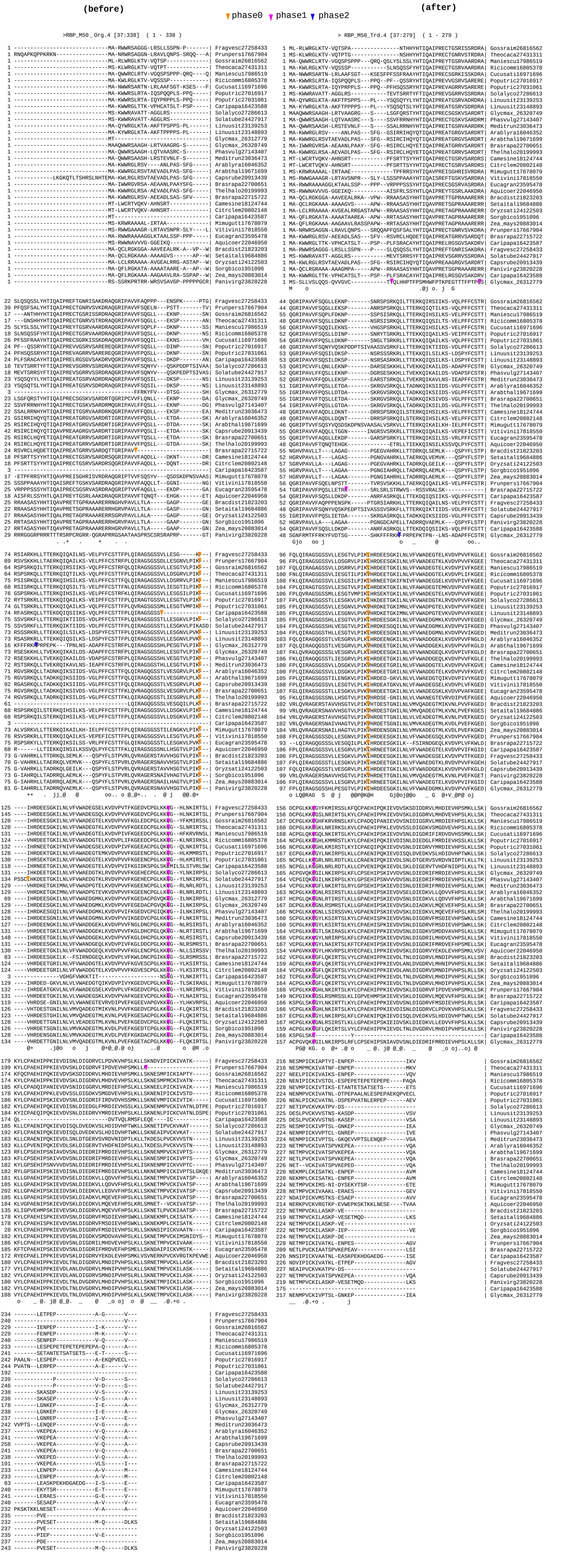

Fig. S1E: An example of GSA-MPSA

(before) ▼phase0 ▼phase1 ▼phase2 (after)

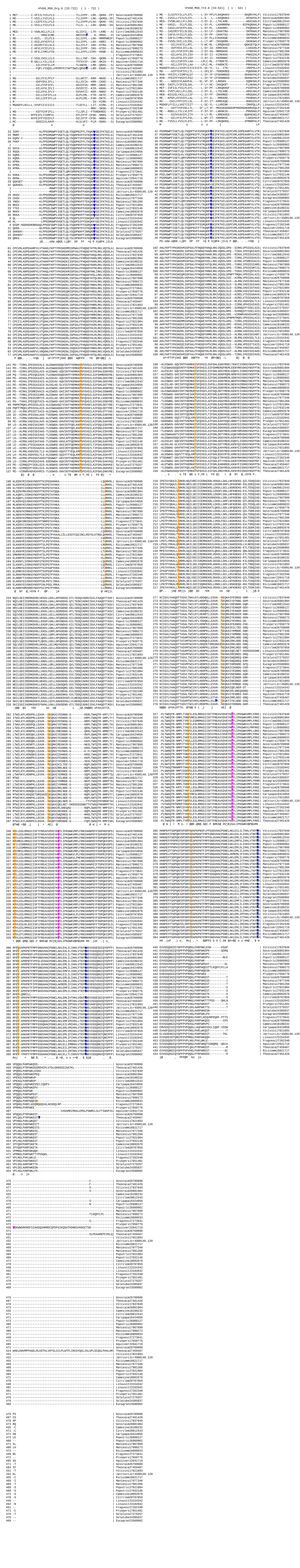

Fig. S1F: An example of GSA-MPSA

▼ phase0 ▼ phase1 ▼ phase2

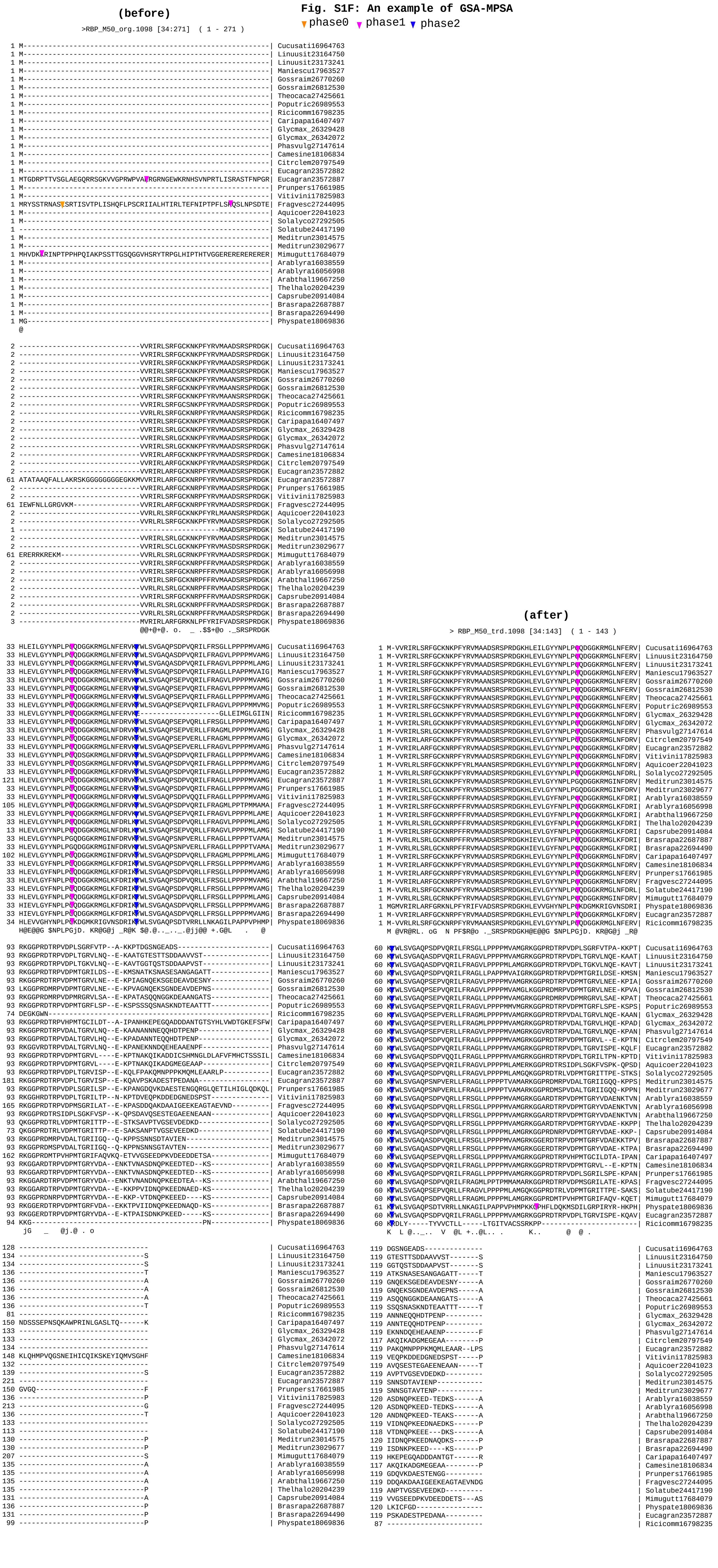

Supplement: Additional file 1: Figure S1 — Examples of GSA-MPSA before and after iterative refinement. Intron positions are indicated by triangles: phase 0 (orange), phase 1 (magenta), and phase 2 (blue). A, C, and E show the results from P450 clusters 1, 4, and 8, respectively, and B, D, and F show the results from ribosomal protein clusters 1, 4, and 1098, respectively, with MaxCluster = 50. For each cluster, CR-M1-PR refinement was performed. [file 1471-2105-15-189-S1.pdf]
