# Supplementary material for: Assessment and refinement of eukaryotic gene structure prediction with gene-structure-aware multiple protein sequence alignment
Source: BMC Bioinformatics. 2014 Jun 14;15:189. doi: 10.1186/1471-2105-15-189 (PMC4065584; doi:10.1186/1471-2105-15-189)
Supplement: Additional file 2: Table S1 — EST-based assessment of gene prediction. NoEST: number of ESTs that overlap with the genic regions of P450 (A, C, E) or ribosomal protein (B, D, F) genes before (A, B) or after (C, D) two-step filtering, and after CR-M1-PR refinement (E, F). NoIntR: total number of introns supported by the ESTs that overlap with the predicted genic regions. NoIntQ: total number of introns in the predicted genes that overlap with at least one of EST mapped areas. IntTP: number of predicted introns that have the same genomic coordinates at the both ends as at least one EST-supported intron. IntFP: number of predicted introns whose genomic regions are assigned as exonic by the EST mapping. IntFN: number of EST-supported introns whose genomic regions are assigned as exonic by prediction. JuncTP: number of predicted exon-intron junctions that have the same genomic coordinates as at least one EST-supported intron. IntHS_TP: number of homology-supported true positive introns. For raw data, IntSn = 100xIntTP/NoIntR, IntSp = 100xIntTP/NoIntQ, JncSn = 50xJncTP/NoIntR, JncSp = 50xJncTP/NoIntQ, FPR = 100xIntFP/NoIntQ, and FNR = 100xIntFN/NoIntR. After correction for homology-supported introns, IntTP, JncTP, and IntFP are replaced by (IntTP + IntHS_TP), (JncTP + 2xIntHS_TP), and (IntFP-IntHS_TP), respectively. Table S2: Manual assessment of peach (A) and maize P450 (B) P450 genes. Seemingly correct and nearly correct predictions are indicated by the letter “C” and “c”, respectively. Near correct prediction implies that either the translational initiation site is ambiguous or the gene contains a frame shift or a premature termination codon within a predicted exon but otherwise looks intact. The letters “E”, “U”, “P”, and “F” respectively indicate erroneous prediction, uncertain prediction, apparent pseudogene, and gene fragment, respectively. Table S3: List of resources of sequences and annotations. An asterisk indicates that P450 genes of that species were once manually curated by one of the aut [file 1471-2105-15-189-S2.pdf]

Table S1A Assessment by EST mapping (P450 all original)

| Species | NoEST | NoIntR | NoIntQ | IntTP | IntFP | IntFN | JuncTP | IntHS_TP |
|---|---|---|---|---|---|---|---|---|
| Aquicoer | 144 | 385 | 389 | 367 | 6 | 1 | 751 | 1 |
| Arablyra | 79 | 163 | 171 | 153 | 8 | 0 | 312 | 5 |
| Arabthal | 239 | 658 | 659 | 650 | 3 | 2 | 1305 | 1 |
| Bracdist | 64 | 208 | 209 | 204 | 2 | 1 | 411 | 2 |
| Brasrapa | 80 | 171 | 174 | 162 | 5 | 0 | 331 | 3 |
| Camesine | 38 | 173 | 173 | 170 | 0 | 0 | 342 | 0 |
| Caripapa | 30 | 59 | 61 | 56 | 2 | 0 | 114 | 2 |
| Chlarein | 15 | 101 | 105 | 100 | 1 | 0 | 200 | 0 |
| Chlovulg | 17 | 67 | 67 | 64 | 0 | 0 | 131 | 0 |
| Citrclem | 95 | 169 | 175 | 167 | 6 | 1 | 335 | 3 |
| Coccsube | 15 | 61 | 62 | 60 | 0 | 0 | 122 | 0 |
| Cucusati | 174 | 535 | 548 | 514 | 15 | 3 | 1045 | 4 |
| Eucagran | 54 | 72 | 74 | 68 | 2 | 0 | 140 | 2 |
| Fragvesc | 187 | 572 | 587 | 510 | 16 | 3 | 1066 | 5 |
| Glycmax_ | 234 | 708 | 720 | 700 | 10 | 0 | 1407 | 2 |
| Gossraim | 139 | 361 | 370 | 344 | 15 | 3 | 702 | 10 |
| Jatrcurc | 28 | 50 | 54 | 47 | 3 | 0 | 98 | 1 |
| Lotujapo | 11 | 21 | 21 | 20 | 0 | 0 | 40 | 0 |
| Maniescu | 58 | 102 | 106 | 100 | 3 | 0 | 201 | 2 |
| Meditrun | 109 | 328 | 330 | 325 | 4 | 1 | 650 | 2 |
| Micrpusi | 1 | 1 | 1 | 1 | 0 | 0 | 2 | 0 |
| Mimugutt | 96 | 223 | 230 | 219 | 9 | 2 | 439 | 0 |
| Oryzsati | 260 | 699 | 703 | 681 | 5 | 2 | 1375 | 3 |
| Ostrluci | 2 | 2 | 2 | 2 | 0 | 0 | 4 | 0 |
| Phasvulg | 62 | 155 | 157 | 152 | 3 | 0 | 306 | 1 |
| Physpate | 68 | 240 | 242 | 235 | 2 | 1 | 471 | 0 |
| Poputric | 105 | 350 | 342 | 321 | 7 | 10 | 656 | 2 |
| Prunpers | 38 | 65 | 75 | 65 | 10 | 0 | 130 | 9 |
| Ricicomm | 205 | 565 | 565 | 563 | 1 | 1 | 1126 | 0 |
| Selamoel | 52 | 169 | 169 | 167 | 1 | 1 | 335 | 0 |
| Solalyco | 123 | 302 | 307 | 296 | 7 | 2 | 596 | 6 |
| Solatube | 136 | 248 | 249 | 235 | 7 | 6 | 478 | 3 |
| Sorgbico | 138 | 333 | 334 | 332 | 1 | 0 | 664 | 0 |
| Thelhalo | 35 | 93 | 97 | 89 | 3 | 0 | 182 | 2 |
| Theocaca | 82 | 172 | 176 | 164 | 5 | 0 | 334 | 2 |
| Vitivini | 195 | 506 | 644 | 485 | 143 | 3 | 978 | 22 |
| Volvcart | 8 | 35 | 34 | 31 | 0 | 1 | 65 | 0 |
| Zea_mays | 213 | 550 | 553 | 539 | 6 | 5 | 1080 | 3 |
| Total | 3629 | 9672 | 9935 | 9358 | 311 | 49 | 18924 | 98 |

| | Raw | with HS_TP |
|---|---|---|
| IntSn | 98 | 98.54545 |
| IntSp | 97.46835 | 98.01085 |
| JncSn | 98.18182 | 98.72727 |
| JncSp | 97.64919 | 98.19168 |
| FPR | 3.130347 | 2.143936 |
| FNR | 0.506617 | 0.506617 |

Table S1B Assessment by EST mapping (RBP all original)

| Species | NoEST | NoIntR | NoIntQ | IntTP | IntFP | IntFN | JuncTP | IntHS_TP |
|---|---|---|---|---|---|---|---|---|
| Aquicoer | 205 | 680 | 692 | 656 | 16 | 3 | 1331 | 14 |
| Arablyra | 263 | 740 | 755 | 711 | 7 | 0 | 1434 | 3 |
| Arabthal | 395 | 1090 | 1099 | 1078 | 5 | 1 | 2169 | 3 |
| Bracdist | 339 | 983 | 990 | 963 | 5 | 0 | 1943 | 2 |
| Brasrapa | 376 | 1012 | 1018 | 981 | 13 | 5 | 1986 | 11 |
| Camesine | 208 | 528 | 527 | 500 | 4 | 5 | 1020 | 0 |
| Caripapa | 177 | 466 | 477 | 455 | 9 | 0 | 920 | 3 |
| Chlarein | 121 | 428 | 427 | 425 | 0 | 0 | 852 | 0 |
| Chlovulg | 116 | 394 | 392 | 386 | 0 | 0 | 778 | 0 |
| Citrclem | 254 | 706 | 707 | 689 | 4 | 1 | 1394 | 3 |
| Coccsube | 90 | 305 | 304 | 300 | 0 | 0 | 603 | 0 |
| Cucusati | 389 | 1147 | 1145 | 1102 | 5 | 2 | 2246 | 2 |
| Eucagran | 283 | 796 | 802 | 731 | 11 | 3 | 1508 | 6 |
| Fragvesc | 200 | 592 | 603 | 557 | 11 | 1 | 1141 | 1 |
| Glycmax_ | 657 | 1978 | 1994 | 1945 | 16 | 1 | 3915 | 4 |
| Gossraim | 406 | 1209 | 1213 | 1107 | 15 | 4 | 2287 | 11 |
| Maniescu | 336 | 991 | 997 | 951 | 9 | 1 | 1934 | 5 |
| Meditrun | 209 | 613 | 621 | 609 | 5 | 0 | 1224 | 2 |
| Micrpusi | 63 | 106 | 109 | 105 | 3 | 0 | 210 | 3 |
| Micrrcc2 | 65 | 103 | 102 | 96 | 0 | 1 | 194 | 0 |
| Mimugutt | 279 | 880 | 884 | 874 | 4 | 0 | 1754 | 1 |
| Oryzsati | 334 | 966 | 976 | 950 | 4 | 0 | 1918 | 3 |
| Ostrluci | 74 | 102 | 102 | 101 | 0 | 0 | 202 | 0 |
| Ostrrcc8 | 28 | 38 | 43 | 36 | 5 | 0 | 73 | 5 |
| Phasvulg | 263 | 805 | 810 | 801 | 5 | 0 | 1606 | 0 |
| Physpate | 466 | 1372 | 1386 | 1359 | 11 | 0 | 2732 | 4 |
| Poputric | 659 | 2044 | 2031 | 1923 | 22 | 13 | 3928 | 6 |
| Prunpers | 199 | 570 | 570 | 566 | 1 | 0 | 1135 | 0 |
| Ricicomm | 235 | 713 | 713 | 712 | 0 | 0 | 1424 | 0 |
| Selamoel | 91 | 268 | 268 | 263 | 1 | 1 | 529 | 1 |
| Solalyco | 271 | 844 | 852 | 825 | 8 | 0 | 1668 | 5 |
| Solatube | 313 | 918 | 914 | 889 | 2 | 4 | 1799 | 1 |
| Sorgbico | 280 | 824 | 828 | 819 | 4 | 0 | 1642 | 1 |
| Thelhalo | 216 | 608 | 608 | 598 | 2 | 1 | 1204 | 2 |
| Theocaca | 286 | 804 | 810 | 768 | 6 | 2 | 1566 | 3 |
| Vitivini | 236 | 680 | 720 | 671 | 38 | 0 | 1352 | 10 |
| Volvcart | 122 | 419 | 421 | 414 | 1 | 0 | 834 | 0 |
| Zea_mays | 765 | 1976 | 1975 | 1892 | 18 | 7 | 3833 | 13 |
| Total | 10269 | 29698 | 29885 | 28808 | 270 | 56 | 58288 | 128 |

| | Raw | with HS_TP |
|---|---|---|
| IntSn | 95.74899 | 96.40688 |
| IntSp | 95.79747 | 96.4557 |
| JncSn | 96.98887 | 97.64676 |
| JncSp | 97.03797 | 97.6962 |
| FPR | 0.903463 | 0.475155 |
| FNR | 0.188565 | 0.188565 |

Table S1C Assessment by EST mapping (P450 filtered original)

| Species | NoEST | NoIntR | NoIntQ | IntTP | IntFP | IntFN | JuncTP | IntHS_FP |
|---|---|---|---|---|---|---|---|---|
| Aquicoer | 89 | 204 | 208 | 199 | 5 | 1 | 402 | 1 |
| Arablyra | 74 | 159 | 167 | 149 | 8 | 0 | 304 | 5 |
| Arabthal | 193 | 508 | 507 | 503 | 1 | 2 | 1009 | 1 |
| Bracdist | 52 | 153 | 154 | 152 | 1 | 0 | 305 | 0 |
| Brasrapa | 72 | 162 | 165 | 155 | 5 | 0 | 315 | 3 |
| Camesine | 11 | 41 | 41 | 41 | 0 | 0 | 82 | 0 |
| Caripapa | 25 | 51 | 53 | 49 | 2 | 0 | 100 | 2 |
| Chlarein | 13 | 89 | 93 | 88 | 1 | 0 | 176 | 0 |
| Chlovulg | 11 | 46 | 46 | 45 | 0 | 0 | 91 | 0 |
| Citrclem | 72 | 123 | 127 | 121 | 4 | 1 | 243 | 2 |
| Coccsube | 10 | 44 | 45 | 43 | 0 | 0 | 88 | 0 |
| Cucusati | 147 | 405 | 413 | 390 | 9 | 2 | 793 | 4 |
| Eucagran | 39 | 56 | 56 | 55 | 0 | 0 | 111 | 0 |
| Fragvesc | 135 | 455 | 462 | 406 | 10 | 1 | 849 | 5 |
| Glycmax_ | 186 | 532 | 538 | 528 | 5 | 0 | 1060 | 1 |
| Gossraim | 80 | 162 | 169 | 158 | 9 | 2 | 318 | 6 |
| Jatrcurc | 19 | 36 | 40 | 34 | 3 | 0 | 71 | 1 |
| Lotujapo | 10 | 18 | 18 | 17 | 0 | 0 | 34 | 0 |
| Maniescu | 42 | 72 | 75 | 70 | 2 | 0 | 141 | 2 |
| Meditrun | 92 | 271 | 271 | 268 | 2 | 1 | 536 | 2 |
| Micrpusi | 1 | 1 | 1 | 1 | 0 | 0 | 2 | 0 |
| Mimugutt | 80 | 183 | 191 | 181 | 9 | 1 | 363 | 0 |
| Oryzsati | 195 | 497 | 503 | 487 | 5 | 1 | 983 | 3 |
| Ostrluci | 1 | 1 | 1 | 1 | 0 | 0 | 2 | 0 |
| Phasvulg | 53 | 128 | 131 | 127 | 3 | 0 | 254 | 1 |
| Physpate | 43 | 147 | 147 | 143 | 0 | 1 | 287 | 0 |
| Poputric | 51 | 136 | 137 | 130 | 5 | 4 | 262 | 1 |
| Prunpers | 36 | 64 | 73 | 64 | 9 | 0 | 128 | 9 |
| Ricicomm | 143 | 454 | 454 | 454 | 0 | 0 | 908 | 0 |
| Selamoel | 40 | 136 | 136 | 135 | 0 | 0 | 271 | 0 |
| Solalyco | 117 | 278 | 282 | 272 | 6 | 2 | 548 | 6 |
| Solatube | 77 | 165 | 165 | 159 | 2 | 3 | 322 | 2 |
| Sorgbico | 121 | 279 | 279 | 278 | 0 | 0 | 556 | 0 |
| Thelhalo | 31 | 82 | 84 | 80 | 1 | 0 | 162 | 0 |
| Theocaca | 48 | 99 | 100 | 97 | 1 | 0 | 196 | 0 |
| Vitivini | 119 | 371 | 443 | 362 | 72 | 0 | 728 | 22 |
| Volvcart | 5 | 24 | 23 | 20 | 0 | 1 | 43 | 0 |
| Zea_mays | 152 | 403 | 408 | 398 | 5 | 2 | 798 | 3 |
| Total | 2685 | 7035 | 7206 | 6860 | 185 | 25 | 13841 | 82 |

| | Raw | 2ith HS_TP |
|---|---|---|
| IntSn | 98.75931 | 99.50372 |
| IntSp | 97.54902 | 98.28431 |
| JncSn | 99.00744 | 99.75186 |
| JncSp | 97.79412 | 98.52941 |
| FPR | 2.567305 | 1.429364 |
| FNR | 0.355366 | 0.355366 |

Table S1D Assessment by EST mapping (RBP filtered original)

| Species | NoEST | NoIntR | NoIntQ | IntTP | IntFP | IntFN | JuncTP | IntHS_TP |
|---|---|---|---|---|---|---|---|---|
| Aquicoer | 114 | 372 | 379 | 365 | 9 | 2 | 734 | 9 |
| Arablyra | 262 | 738 | 753 | 709 | 7 | 0 | 1430 | 3 |
| Arabthal | 288 | 811 | 818 | 805 | 3 | 1 | 1618 | 2 |
| Bracdist | 244 | 719 | 724 | 709 | 4 | 0 | 1428 | 1 |
| Brasrapa | 372 | 1006 | 1011 | 975 | 12 | 5 | 1974 | 11 |
| Camesine | 99 | 260 | 257 | 249 | 1 | 4 | 505 | 0 |
| Caripapa | 162 | 423 | 434 | 413 | 9 | 0 | 835 | 3 |
| Chlarein | 97 | 348 | 348 | 347 | 0 | 0 | 695 | 0 |
| Chlovulg | 104 | 352 | 352 | 350 | 0 | 0 | 702 | 0 |
| Citrclem | 166 | 471 | 473 | 466 | 4 | 1 | 936 | 3 |
| Coccsube | 83 | 281 | 281 | 278 | 0 | 0 | 558 | 0 |
| Cucusati | 236 | 702 | 704 | 685 | 4 | 1 | 1387 | 1 |
| Eucagran | 169 | 460 | 460 | 439 | 2 | 2 | 893 | 0 |
| Fragvesc | 188 | 554 | 564 | 529 | 7 | 1 | 1080 | 1 |
| Glycmax_ | 484 | 1501 | 1515 | 1475 | 14 | 1 | 2970 | 4 |
| Gossraim | 184 | 548 | 550 | 516 | 8 | 4 | 1053 | 6 |
| Maniescu | 242 | 731 | 732 | 718 | 3 | 1 | 1445 | 2 |
| Meditrun | 178 | 536 | 544 | 532 | 5 | 0 | 1070 | 2 |
| Micrpusi | 61 | 102 | 105 | 101 | 3 | 0 | 202 | 3 |
| Micrrcc2 | 61 | 96 | 95 | 89 | 0 | 1 | 180 | 0 |
| Mimugutt | 219 | 678 | 683 | 676 | 4 | 0 | 1354 | 1 |
| Oryzsati | 263 | 777 | 785 | 767 | 1 | 0 | 1549 | 1 |
| Ostrluci | 69 | 94 | 94 | 93 | 0 | 0 | 186 | 0 |
| Ostrrcc8 | 26 | 35 | 40 | 33 | 5 | 0 | 67 | 5 |
| Phasvulg | 237 | 739 | 744 | 736 | 5 | 0 | 1475 | 0 |
| Physpate | 385 | 1115 | 1124 | 1105 | 6 | 0 | 2222 | 1 |
| Poputric | 346 | 1079 | 1069 | 1033 | 5 | 10 | 2096 | 2 |
| Prunpers | 184 | 529 | 528 | 526 | 0 | 0 | 1054 | 0 |
| Ricicomm | 229 | 688 | 688 | 687 | 0 | 0 | 1374 | 0 |
| Selamoel | 87 | 257 | 257 | 253 | 1 | 1 | 508 | 1 |
| Solalyco | 269 | 833 | 841 | 814 | 8 | 0 | 1646 | 5 |
| Solatube | 246 | 743 | 741 | 729 | 2 | 2 | 1468 | 0 |
| Sorgbico | 256 | 764 | 768 | 762 | 3 | 0 | 1527 | 1 |
| Thelhalo | 181 | 514 | 514 | 509 | 2 | 1 | 1021 | 2 |
| Theocaca | 198 | 574 | 581 | 554 | 4 | 1 | 1126 | 2 |
| Vitivini | 227 | 661 | 700 | 653 | 37 | 0 | 1315 | 9 |
| Volvcart | 94 | 318 | 318 | 314 | 0 | 0 | 632 | 0 |
| Zea_mays | 459 | 1250 | 1239 | 1196 | 3 | 6 | 2426 | 2 |
| Total | 7769 | 22659 | 22813 | 22190 | 181 | 45 | 44741 | 83 |

| | Raw | with HS_TP |
|---|---|---|
| IntSn | 97.93018 | 98.29648 |
| IntSp | 97.2691 | 97.63293 |
| JncSn | 98.72678 | 99.09308 |
| JncSp | 98.06032 | 98.42414 |
| FPR | 0.793407 | 0.42958 |
| FNR | 0.198597 | 0.198597 |

Table S1E Assessment by EST mapping (P450 refined)

| Species | NoEST | NoIntR | NoIntQ | IntTP | IntFP | IntFN | JuncTP | IntHS_TP |
|---|---|---|---|---|---|---|---|---|
| Aquicoer | 86 | 201 | 204 | 197 | 3 | 1 | 398 | 1 |
| Arablyra | 73 | 157 | 159 | 151 | 2 | 1 | 307 | 1 |
| Arabthal | 193 | 502 | 516 | 488 | 5 | 1 | 987 | 5 |
| Bracdist | 53 | 150 | 152 | 148 | 1 | 0 | 298 | 2 |
| Brasrapa | 68 | 161 | 167 | 158 | 3 | 0 | 318 | 2 |
| Camesine | 11 | 42 | 41 | 40 | 0 | 1 | 81 | 0 |
| Caripapa | 25 | 51 | 54 | 48 | 3 | 0 | 99 | 2 |
| Chlarein | 11 | 81 | 81 | 81 | 0 | 0 | 162 | 0 |
| Chlovulg | 11 | 46 | 46 | 46 | 0 | 0 | 92 | 0 |
| Citrclem | 71 | 121 | 124 | 120 | 3 | 0 | 241 | 3 |
| Coccsube | 10 | 43 | 43 | 43 | 0 | 0 | 86 | 0 |
| Cucusati | 144 | 407 | 412 | 395 | 5 | 3 | 800 | 4 |
| Eucagran | 37 | 56 | 56 | 55 | 0 | 0 | 111 | 0 |
| Fragvesc | 138 | 455 | 457 | 448 | 0 | 0 | 902 | 0 |
| Glycmax_ | 186 | 532 | 537 | 526 | 4 | 0 | 1058 | 0 |
| Gossraim | 80 | 162 | 171 | 162 | 9 | 0 | 324 | 8 |
| Jatrcurc | 17 | 34 | 36 | 34 | 2 | 0 | 68 | 0 |
| Lotujapo | 10 | 18 | 19 | 16 | 0 | 0 | 34 | 0 |
| Maniescu | 42 | 72 | 75 | 71 | 2 | 0 | 142 | 1 |
| Meditrun | 93 | 256 | 266 | 245 | 12 | 1 | 497 | 5 |
| Micrpusi | 1 | 1 | 1 | 1 | 0 | 0 | 2 | 0 |
| Mimugutt | 77 | 184 | 193 | 181 | 2 | 1 | 364 | 0 |
| Oryzsati | 195 | 494 | 495 | 476 | 7 | 1 | 961 | 3 |
| Ostrluci | 1 | 1 | 2 | 1 | 1 | 0 | 2 | 0 |
| Phasvulg | 53 | 128 | 130 | 127 | 2 | 0 | 254 | 1 |
| Physpate | 45 | 145 | 149 | 134 | 4 | 1 | 273 | 1 |
| Poputric | 50 | 137 | 135 | 129 | 2 | 4 | 262 | 0 |
| Prunpers | 35 | 62 | 71 | 62 | 9 | 0 | 124 | 9 |
| Ricicomm | 142 | 437 | 434 | 418 | 2 | 6 | 844 | 1 |
| Selamoel | 37 | 122 | 123 | 119 | 0 | 0 | 242 | 0 |
| Solalyco | 117 | 277 | 283 | 272 | 8 | 2 | 547 | 7 |
| Solatube | 76 | 171 | 169 | 167 | 0 | 2 | 335 | 0 |
| Sorgbico | 120 | 276 | 275 | 272 | 0 | 1 | 547 | 0 |
| Thelhalo | 31 | 81 | 83 | 79 | 1 | 0 | 161 | 1 |
| Theocaca | 46 | 96 | 97 | 93 | 1 | 0 | 189 | 1 |
| Vitivini | 119 | 368 | 369 | 367 | 2 | 1 | 734 | 1 |
| Volvcart | 5 | 24 | 24 | 20 | 1 | 1 | 42 | 0 |
| Zea_mays | 150 | 403 | 410 | 400 | 8 | 1 | 802 | 3 |
| Total | 2659 | 6954 | 7059 | 6790 | 104 | 29 | 13690 | 62 |

| | Raw | with HS_TP |
|---|---|---|
| IntSn | 97.64165 | 98.53322 |
| IntSp | 96.18926 | 97.06757 |
| JncSn | 98.43256 | 99.32413 |
| JncSp | 96.96841 | 97.84672 |
| FPR | 1.473297 | 0.594985 |
| FNR | 0.417026 | 0.417026 |

Table S1F Assessment by EST mapping (RBP refined)

| Species | NoEST | NoIntR | NoIntQ | IntTP | IntFP | IntFN | JuncTP | IntHS_TP |
|---|---|---|---|---|---|---|---|---|
| Aquicoer | 111 | 366 | 377 | 361 | 12 | 0 | 725 | 10 |
| Arablyra | 262 | 738 | 749 | 731 | 6 | 0 | 1467 | 4 |
| Arabthal | 289 | 803 | 809 | 797 | 7 | 2 | 1597 | 5 |
| Bracdist | 242 | 709 | 711 | 706 | 1 | 0 | 1416 | 0 |
| Brasrapa | 374 | 1027 | 1042 | 1013 | 13 | 1 | 2038 | 11 |
| Camesine | 99 | 259 | 259 | 245 | 1 | 1 | 496 | 1 |
| Caripapa | 162 | 426 | 436 | 419 | 10 | 0 | 844 | 3 |
| Chlarein | 97 | 344 | 342 | 341 | 0 | 0 | 684 | 0 |
| Chlovulg | 104 | 359 | 355 | 351 | 0 | 0 | 708 | 0 |
| Citrclem | 165 | 473 | 479 | 470 | 6 | 1 | 943 | 2 |
| Coccsube | 83 | 283 | 279 | 270 | 0 | 0 | 551 | 0 |
| Cucusati | 236 | 712 | 718 | 686 | 5 | 1 | 1389 | 3 |
| Eucagran | 168 | 448 | 453 | 438 | 3 | 0 | 887 | 1 |
| Fragvesc | 185 | 543 | 548 | 533 | 6 | 0 | 1070 | 2 |
| Glycmax_ | 495 | 1528 | 1551 | 1482 | 18 | 0 | 3002 | 10 |
| Gossraim | 184 | 549 | 559 | 538 | 9 | 1 | 1083 | 8 |
| Maniescu | 240 | 717 | 720 | 705 | 8 | 0 | 1418 | 4 |
| Meditrun | 170 | 478 | 485 | 471 | 10 | 0 | 946 | 6 |
| Micrpusi | 58 | 100 | 104 | 96 | 4 | 0 | 196 | 3 |
| Micrrcc2 | 61 | 94 | 98 | 93 | 4 | 0 | 186 | 3 |
| Mimugutt | 217 | 667 | 675 | 655 | 6 | 0 | 1318 | 2 |
| Oryzsati | 271 | 787 | 810 | 774 | 16 | 0 | 1559 | 9 |
| Ostrluci | 70 | 92 | 96 | 88 | 5 | 2 | 178 | 3 |
| Ostrrcc8 | 26 | 38 | 45 | 37 | 6 | 0 | 76 | 5 |
| Phasvulg | 239 | 727 | 733 | 721 | 8 | 0 | 1447 | 3 |
| Physpate | 395 | 1105 | 1140 | 1072 | 30 | 0 | 2174 | 10 |
| Poputric | 344 | 1055 | 1059 | 1039 | 9 | 3 | 2089 | 6 |
| Prunpers | 183 | 518 | 519 | 513 | 0 | 0 | 1030 | 0 |
| Ricicomm | 221 | 639 | 638 | 610 | 2 | 2 | 1240 | 2 |
| Selamoel | 95 | 273 | 279 | 265 | 7 | 0 | 534 | 4 |
| Solalyco | 268 | 812 | 814 | 798 | 5 | 0 | 1607 | 4 |
| Solatube | 252 | 757 | 761 | 748 | 7 | 2 | 1501 | 3 |
| Sorgbico | 257 | 753 | 762 | 743 | 7 | 0 | 1493 | 5 |
| Thelhalo | 181 | 511 | 514 | 509 | 2 | 0 | 1021 | 2 |
| Theocaca | 201 | 569 | 571 | 557 | 3 | 0 | 1124 | 1 |
| Vitivini | 216 | 654 | 660 | 644 | 8 | 0 | 1296 | 3 |
| Volvcart | 94 | 318 | 318 | 315 | 0 | 0 | 632 | 0 |
| Zea_mays | 470 | 1251 | 1271 | 1187 | 27 | 3 | 2421 | 12 |
| Total | 7785 | 22482 | 22739 | 22021 | 271 | 19 | 44386 | 150 |

| | Raw | with HS_TP |
|---|---|---|
| IntSn | 97.94947 | 98.61667 |
| IntSp | 96.84243 | 97.50209 |
| JncSn | 98.71453 | 99.38173 |
| JncSp | 97.59884 | 98.2585 |
| FPR | 1.191785 | 0.532125 |
| FNR | 0.084512 | 0.084512 |

Table S2A Mnual Assesssment of Peach P450 Genes

| KOG-PID | PeachID | Subfamily | Chrom. | Strand | Cluster | Phytozome | Comment | Refgs | Comment |
|---|---|---|---|---|---|---|---|---|---|
| 17641033 | ppa005745m | CYP88A | 5 | − | 16 | E | short N | C | |
| 17641081 | ppa026875m | CYP715A | 2 | + | 394 | C | | C | |
| 17641236 | | | 5 | + | 0 | F | 173aa | F | |
| 17641348 | ppa004078m | CYP86A | 5 | − | 420 | C | | C | |
| 17641366 | ppa023692m | CYP734A | 1 | + | 374 | C | | C | |
| 17641500 | ppb017968m | | 1 | + | 0 | F | 243aa | F | G? |
| 17641532 | ppa002280m | reductase | 1 | + | 480 | X | | X | |
| 17641582 | ppa018300m | | 4 | − | 0 | F | 194aa | F | |
| 17641722 | ppa016513m | CYP81B | 1 | + | 249 | E | short C | C | |
| 17641732 | ppa004569m | CYP82G | 8 | − | 222 | E | extra I | C | |
| 17642122 | ppa004592m | CYP94C | 6 | − | 437 | C | | C | |
| 17642372 | ppa019319m | CYP94A | 8 | − | 447 | E | non start codon | C | |
| 17642385 | ppa017703m | CYP82C | 1 | − | 209 | E | short N | C | |
| 17642457 | ppa023623m | CYP720A | 5 | + | 49 | C | | C | |
| 17642522 | ppa005059m | CYP707A | 5 | + | 4 | C | | C | |
| 17643034 | ppa026777m | CYP734A | 7 | + | 382 | C | | C | |
| 17643059 | ppa026952m | CYP76F | 1 | + | 149 | C | | C | |
| 17643169 | ppa017300m | CYP82C | 3 | − | 211 | C | | C | |
| 17643207 | ppa026370m | CYP77B | 7 | + | 286 | C | | C | |
| 17643213 | ppa003997m | CYP78A | 2 | − | 274 | E | extra I | C | |
| 17643387 | ppa019821m | CYP93A | 7 | + | 225 | C | | C | |
| 17643533 | ppa019694m | | 1 | + | 0 | F | 143aa | F | |
| 17643605 | ppa004444m | CYP94A | 8 | − | 447 | C | | C | |
| 17643882 | ppa027164m | CYP706A | 5 | + | 200 | C | | C | |
| 17643885 | ppa017464m | CYP82C | 1 | − | 215 | E | short N | C | |
| 17643926 | ppa004832m | CYP92A | 7 | − | 173 | E | extra I | c | FS |
| 17644135 | ppa004606m | CYP76F | 5 | − | 149 | C | | C | |
| 17644145 | ppa015588m | CYP734A | 1 | − | 374 | C | | C | |
| 17644410 | ppa017328m | CYP725A | 4 | − | 21 | C | | C | |
| 17644601 | ppb018168m | | 1 | + | 0 | F | 180aa | F | |
| 17644659 | ppa022132m | CYP76F | 1 | − | 149 | C | | C | |
| 17644816 | ppa004443m | CYP76F | 8 | + | 282 | E | many defects | C | |
| 17645100 | ppa023159m | | 1 | + | 0 | F | 99aa | F | |
| 17645137 | ppa017206m | CYP71A | 3 | − | 119 | C | | C | |
| 17645204 | ppa003882m | CYP78A | 5 | − | 276 | C | | C | |
| 17645260 | ppa018724m | CYP734A | 1 | − | 374 | C | | C | |
| 17645473 | ppa024054m | | 5 | + | 0 | F | 243aa | F | |
| 17645509 | ppa020034m | CYP93B | 6 | − | 232 | C | | C | |
| 17645715 | ppa004404m | CYP98A | 1 | + | 163 | C | | C | |
| 17645828 | ppa004496m | CYP714A | 6 | − | 400 | E | short N | C | |
| 17645872 | ppa023495m | CYP82A | 1 | − | 211 | C | | C | |
| 17645874 | ppa004414m | CYP72X | 1 | − | 363 | C | | C | |
| 17645889 | ppa004390m | CYP71B | 1 | − | 123 | C | | C | |
| 17645942 | ppa015526m | CYP72A | 4 | + | 351 | E | extra I | c | FS |
| 17646105 | ppa025666m | CYP716A | 6 | − | 34 | E | extra E | C | |
| 17646117 | ppa018082m | CYP712A | 2 | − | 240 | C | | c | short N 3aa |
| 17646196 | ppa024213m | CYP78A | 1 | − | 271 | C | | C | |
| 17646410 | ppa022359m | CYP704A | 2 | + | 465 | C | | C | |
| 17646594 | ppa019974m | | 2 | + | 465 | P | many defects | P | many defects |
| 17646693 | ppa003980m | CYP89A | 2 | + | 287 | C | | C | |
| 17646705 | ppa003405m | CYP97B | 3 | + | 412 | C | | C | |
| 17646731 | ppa004199m | CYP89A | 2 | + | 287 | C | | C | |
| 17647258 | ppa017795m | CYP82C | 1 | − | 211 | P | short N | P | |
| 17647335 | ppa004034m | CYP86A | 2 | − | 419 | C | | C | |
| 17647395 | ppa004169m | CYP82A | 1 | − | 211 | C | | C | |
| 17647582 | ppa006310m | CYP72A | 4 | + | 351 | E | short N | C | |
| 17647602 | ppa019218m | CYP706A | 5 | + | 200 | E | short N | C | |
| 17647730 | ppa023100m | CYP734A | 1 | − | 374 | E | short N | C | |
| 17647741 | ppa019068m | CYP92A | 2 | + | 188 | C | | C | |
| 17647755 | ppa017688m | CYP71A? | 5 | + | 186 | E | extra I | c | FS |
| 17647775 | ppa018639m | CYP735A | 3 | − | 387 | C | | C | |
| 17647819 | ppa017772m | | 1 | − | 0 | F | 132aa | F | |
| 17647937 | ppa004261m | CYP71A | 5 | − | 119 | C | | C | |
| 17647986 | ppa017053m | | 7 | + | 0 | F | 156aa | F | |
| 17648032 | ppa024382m | CYP87A | 8 | − | 62 | C | | C | |
| 17648033 | ppa005020m | CYP707A | 6 | + | 7 | C | | C | |
| 17648154 | ppa005225m | CYP716A | 4 | − | 37 | C | | C | |
| 17648300 | ppa004999m | CYP716A | 4 | − | 37 | C | | C | |
| 17648302 | ppa018515m | | 6 | + | 0 | F | 180aa | F | |
| 17648355 | ppa014656m | | 1 | + | 0 | F | 151aa | F | |
| 17648436 | ppa1027202m | CYP710A | 4 | − | 470 | c | 7aa short N | C | |
| 17648601 | ppa005322m | CYP706A | 5 | + | 194 | E | short N | C | |

| | | | | | | | | | |
|---|---|---|---|---|---|---|---|---|---|
| 17648621 | ppa005465m | CYP725A | 6 | + | 20 | E | extra I | C | |
| 17648680 | ppa004329m | CYP734A | 1 | − | 374 | C | | C | |
| 17648717 | ppa019886m | CYP81B | 1 | + | 254 | E | extra I | C | |
| 17648736 | ppa024099m | CYP706A | 5 | + | 194 | E | short N | C | |
| 17648820 | ppa018268m | CYP76A | 7 | − | 142 | C | | c | 1aa short N |
| 17648862 | ppa023510m | CYP82C | 8 | + | 223 | C | | C | |
| 17648959 | ppa004947m | CYP704A | 2 | + | 461 | E | extra I | C | |
| 17649004 | ppa023299m | CYP76F | 1 | + | 149 | E | long D | c | FS |
| 17649063 | ppa024021m | CYP81B | 1 | − | 245 | E | short N; extra I | C | |
| 17649198 | ppa005988m | CYP707A | 7 | + | 13 | E | missing E | C | |
| 17649311 | ppa019443m | CYP706A | 5 | + | 200 | E | short N | E | extra I |
| 17649531 | ppa004095m | CYP82G | 8 | − | 222 | C | | C | |
| 17649549 | ppa022722m | | 1 | + | 249 | P | | P | FS |
| 17649579 | ppa016446m | CYP72A | 1 | − | 351 | C | | C | |
| 17649969 | ppa024360m | CYP90D | 3 | + | 50 | C | | C | |
| 17650111 | ppa004988m | CYP716A | 1 | − | 26 | C | | C | |
| 17650252 | ppa004639m | CYP77F | 1 | + | 149 | C | | C | |
| 17650355 | ppa017329m | CYP76F | 1 | + | 149 | E | extra I | C | |
| 17650379 | ppa016822m | CYP722A | 1 | + | 2 | C | | C | |
| 17650462 | ppa018839m | CYP71D | 3 | + | 78 | c | 3aa short N | C | |
| 17650487 | ppa011895m | | 1 | − | 0 | F | 192aa | F | |
| 17650575 | ppa021608m | | 5 | + | 0 | F | 135aa | F | |
| 17650747 | ppa023634m | | 1 | − | 0 | F | 312aa | F | |
| 17650776 | ppa005226m | CYP707A | 8 | − | 8 | C | | C | |
| 17650839 | | | 4 | − | 0 | F | 74aa | F | |
| 17650968 | ppa004617m | CYP76F | 1 | + | 149 | C | | C | |
| 17650973 | ppa005500m | CYP725A | 6 | + | 20 | E | short N | C | |
| 17651200 | ppa026664m | CYP89A | 5 | − | 304 | C | | C | |
| 17651265 | ppa022977m | CYP71A | 4 | − | 106 | P | many defects | P | some defects |
| 17651273 | ppa026995m | | 2 | − | 0 | F | 155aa | F | |
| 17651331 | ppa019918m | | 2 | − | 0 | F | 303aa | F | |
| 17651416 | ppa022312m | CYP93A | 7 | + | 225 | E | missing E x 2 | c | FS |
| 17651466 | ppa016887m | CYP734A | 8 | + | 374 | C | | C | |
| 17651481 | ppa005234m | CYP7074A | 1 | + | 8 | C | | C | |
| 17651573 | ppa021326m | CYP79D | 6 | + | 314 | E | short N | C | |
| 17651808 | ppa019129m | | 6 | − | 0 | F | 187aa | F | |
| 17651873 | ppa016904m | CYP704A | 2 | + | 465 | C | | C | |
| 17651883 | ppa004175m | CYP83A | 1 | − | 211 | C | | C | |
| 17651909 | ppa004271m | CYP72A | 4 | + | 351 | C | | C | |
| 17652064 | ppa004356m | CYP89A | 2 | + | 287 | C | | C | |
| 17652232 | ppa022777m | CYP92A | 7 | + | 173 | C | | C | |
| 17652233 | ppa015528m | | 1 | − | 0 | F | 219aa | F | |
| 17652304 | ppa017406m | | 4 | − | 0 | F | 331aa | F | |
| 17652428 | ppa019048m | CYP92A | 7 | + | 173 | C | | C | |
| 17652470 | ppa015200m | CYP82C | 1 | − | 215 | E | short N | C | |
| 17652600 | | | 1 | − | 0 | F | 102aa | F | |
| 17652612 | ppa026652m | CYP92A | 4 | + | 188 | C | | C | |
| 17652654 | ppa004503m | CYP76F | 2 | − | 149 | C | | C | |
| 17652656 | ppa026874m | | 1 | − | 0 | F | 338aa | F | |
| 17652743 | ppa004664m | CYP92A | 4 | − | 188 | C | | C | |
| 17652852 | ppa027160m | CYP82C | 3 | − | 211 | C | | C | |
| 17652931 | ppa004467m | CYP94D | 5 | − | 450 | C | | C | |
| 17652968 | ppa026124m | CYP704A | 2 | + | 461 | C | | C | |
| 17653004 | | | 8 | + | 0 | F | 178aa | F | |
| 17653014 | ppa016631m | | 6 | − | 0 | F | 380aa | F | G? |
| 17653353 | ppa026596m | | 1 | + | 0 | F | 86aa | F | |
| 17653792 | ppa015856m | CYP724A | 3 | − | 43 | E | short N | C | |
| 17654039 | ppa018745m | CYP87A | 5 | + | 55 | E | extra I | C | |
| 17654058 | ppa004373m | CYP77A | 2 | − | 284 | C | | C | |
| 17654300 | ppa015948m | | 1 | 0 | 0 | F | 130aa | F | |
| 17654330 | ppa012947m | | 5 | − | 0 | F | 148aa | F | |
| 17654341 | ppa021340m | | 6 | − | 0 | F | 185aa | F | |
| 17654437 | ppa003950m | CYP711 | 1 | + | 329 | C | | C | |
| 17654549 | | | 3 | − | 0 | F | 131aa | F | |
| 17654756 | | | 6 | + | 0 | F | 144aa | F | |
| 17654762 | ppa017476m | CYP82C | 1 | − | 215 | F | short N | F | many defects |
| 17654770 | ppa004328m | CYP72B | 1 | + | 374 | C | | C | |
| 17654811 | ppa025865m | | 8 | + | 0 | F | 332aa | F | |
| 17654823 | ppa004332m | CYP71A | 5 | − | 106 | C | | C | |
| 17654861 | ppa017915m | CYP724A | 1 | + | 351 | C | | E | short N |
| 17654864 | ppa018687m | CYP712A | 7 | + | 241 | E | extra E | C | |
| 17654919 | | | 4 | − | 0 | F | 69aa | F | |
| 17654960 | ppa023521m | CYP79A | 7 | − | 313 | E | short N | C | |
| 17655051 | ppa017888m | | 34 | − | 0 | F | 194aa | F | |
| 17655150 | ppa004464m | CYP82C | 1 | − | 211 | U | extra I? | U | extra I? |

| | | | | | | | | | |
|---|---|---|---|---|---|---|---|---|---|
| 17655171 | ppa018666m | CYP71D | 4 | − | 84 | E | short N | C | |
| 17655251 | ppa017849m | | 4 | − | 0 | F | 398aa | F | |
| 17655499 | ppa023262m | | 1 | − | 0 | F | 237aa | F | |
| 17655679 | ppa004343m | CYP71B | 1 | − | 123 | C | | C | |
| 17655766 | ppa018902m | | 6 | + | 0 | F | 72aa | F | |
| 17655807 | ppa024660m | CYP734A | 1 | + | 374 | C | | C | |
| 17655862 | ppa005680m | CYP722A | 7 | − | 3 | E | short N | C | |
| 17655902 | ppa020227m | CYP86A | 6 | − | 418 | E | extended N | C | |
| 17655981 | ppb022807m | | 1 | + | 0 | F | 255aa | F | |
| 17656111 | ppa005937m | CYP88A | 2 | − | 16 | E | short N | C | |
| 17656126 | ppa004319m | CYP734A | 1 | − | 374 | C | | C | |
| 17656205 | ppa003701m | CYP97C | 7 | + | 406 | C | | C | |
| 17656241 | ppa005190m | CYP71A | 4 | + | 106 | E | extra I | C | |
| 17656265 | ppa017252m | CYP94B | 5 | + | 442 | E | short C | C | |
| 17656343 | ppa021443m | | 8 | + | 0 | F | 392aa | F | |
| 17656469 | ppa018047m | | 7 | + | 0 | F | 154aa | F | |
| 17656503 | ppa019849m | CYP706A | 5 | + | 200 | C | | E | short N |
| 17656657 | ppa020167m | | 5 | + | 0 | F | 110aa | F | |
| 17656672 | ppa026438m | CYP92A | 7 | + | 173 | C | | C | |
| 17656771 | ppa004331m | CYP86B | 1 | − | 424 | E | short N | C | |
| 17656776 | ppa020463m | CYP71D | 1 | − | 78 | E | short N | C | |
| 17656959 | ppa004635m | CYP81E | 6 | + | 255 | C | | C | |
| 17657040 | ppa020002m | CYP71A | 5 | − | 121 | C | | C | |
| 17657044 | ppa019660m | CYP706A | 5 | + | 194 | C | | C | |
| 17657054 | ppa021108m | CYP72X | 7 | − | 363 | E | wrong 5'end of E4 | C | |
| 17657067 | ppa025166m | CYP94C | 7 | + | 438 | E | short N | C | |
| 17657069 | ppa004266m | CYP714A | 6 | − | 401 | E | short N | C | |
| 17657151 | ppa003455m | CYP97A | 6 | − | 409 | E | lack E; extra I | C | |
| 17657235 | ppa004406m | CYP98A | 1 | + | 163 | C | | C | |
| 17657264 | ppa005918m | CYP82C | 1 | − | 215 | E | short N | C | |
| 17657289 | ppa004646m | CYP734A | 1 | − | 374 | E | long D | c | FS |
| 17657351 | ppa004565m | CYP90B | 7 | + | 46 | C | | c | 8aa short N |
| 17657563 | ppa019215m | CYP92A | 7 | − | 173 | C | | C | |
| 17657572 | ppa004474m | CYP98A | 1 | + | 163 | C | | C | |
| 17657585 | ppa017288m | | 7 | + | 0 | F | 91aa | F | |
| 17657682 | ppa1027185m | CYP711A | 1 | − | 329 | C | | C | |
| 17657947 | ppa1027205m | CYP714P | 7 | + | 399 | F | many defects | F | many defects |
| 17657952 | ppa014661m | | 4 | − | 0 | F | 211aa | F | |
| 17657992 | ppa007545m | | 8 | + | 0 | F | 364aa | F | |
| 17658323 | ppa004179m | CYP84A | 1 | − | 211 | C | | C | |
| 17658392 | ppa020637m | CYP93A | 7 | + | 225 | C | | C | |
| 17658399 | ppa016050m | CYP82G | 8 | − | 222 | E | short N | C | |
| 17658496 | ppa019666m | CYP87A | 5 | + | 55 | C | | C | |
| 17658543 | ppa023454m | CYP724A | 3 | + | 43 | E | many defects | E | intron position |
| 17658574 | ppa023562m | | 7 | + | 0 | F | 227 | F | |
| 17658874 | | | 1 | − | 0 | F | 84aa | F | |
| 17659036 | ppa016530m | | 4 | + | 0 | F | 194aa | F | |
| 17659037 | ppa004311m | CYP94B | 3 | − | 442 | C | | C | |
| 17659053 | ppa016601m | | 5 | + | 374 | F | | F | many defects |
| 17659061 | ppa017401m | CYP706A | 5 | + | 194 | C | | C | |
| 17659069 | ppa025750m | CYP93B | 6 | + | 232 | C | | C | |
| 17659173 | ppa024556m | CYP71D | 4 | − | 84 | c | 7aa short N | C | |
| 17659209 | ppa019787m | CYP711 | 1 | − | 329 | C | | C | |
| 17659229 | ppa016770m | CYP72A | 4 | + | 351 | E | extra I | C | |
| 17659467 | ppa026558m | CYP88A | 2 | + | 16 | E | extra E | E | extra E near C |
| 17659547 | ppa025126m | CYP71A | 4 | + | 106 | E | extra I x 2 | C | |
| 17659643 | ppa015701m | CYP714A | 2 | + | 399 | c | 4aa short N | C | |
| 17659755 | ppa018530m | CYP714A | 2 | + | 399 | C | | C | |
| 17659866 | ppa014572m | CYP92A | 4 | − | 188 | C | | C | |
| 17659957 | ppa023488m | CYP725A | 4 | − | 21 | C | | C | |
| 17660198 | ppa004449m | CYP94A | 8 | − | 447 | C | | C | |
| 17660318 | ppa019965m | CYP81E | 6 | + | 255 | C | | C | |
| 17660710 | ppa020499m | | 7 | + | 0 | F | 361aa | F | |
| 17660794 | ppa004910m | CYP88A | 2 | + | 16 | C | | C | |
| 17660878 | ppa017951m | | 4 | + | 0 | F | 318aa | F | |
| 17661119 | ppa025196m | CYP735A | 1 | + | 374 | C | | C | |
| 17661131 | ppa020113m | | 2 | − | 0 | F | 141aa | F | |
| 17661246 | ppa004083m | CYP78A | 5 | + | 272 | C | | C | |
| 17661320 | ppa004539m | CYP721 | 1 | + | 385 | C | | C | |
| 17661453 | ppa008535m | | 5 | + | 0 | F | 328aa | F | |
| 17661551 | ppa004268m | CYP714A | 2 | − | 396 | C | | C | |
| 17661757 | ppa004281m | CYP706A | 5 | + | 194 | C | | C | |
| 17661806 | ppa016042m | | 1 | − | 0 | F | 287aa | F | |
| 17662015 | ppa021901m | | 1 | + | 374 | P | long D | P | long D |
| 17662021 | ppa026628m | CYP92A | 4 | − | 188 | C | | C | |

| | | | | | | | | | |
|---|---|---|---|---|---|---|---|---|---|
| 17662158 | ppa004341m | CYP714A | 6 | − | 400 | C | | C | |
| 17662207 | ppa021147m | CYP76F | 1 | + | 149 | E | short N | C | |
| 17662274 | ppa016912m | CYP82C | 1 | − | 215 | E | short N | C | |
| 17662330 | ppa024670m | CYP71A | 5 | − | 121 | C | | C | |
| 17662402 | ppa005870m | CYP71D | 1 | − | 78 | E | short N | C | |
| 17662502 | ppa024825m | | 5 | + | 0 | F | 397aa | F | |
| 17662527 | ppa025974m | | 2 | − | 0 | F | 315a | F | |
| 17662779 | ppa014596m | CYP92A | 7 | − | 173 | E | extra I | c | FS |
| 17662905 | | | 6 | − | 0 | F | 117aa | F | |
| 17662935 | ppa003468m | CYP727A | 2 | + | 469 | C | | C | |
| 17662955 | ppa016466m | CYP82G | 8 | − | 62 | E | wrong 5'end of E4 | C | |
| 17663193 | ppa024661m | CYP92A | 7 | + | 225 | C | | C | |
| 17663261 | ppa024029m | CYP715A | 2 | + | 394 | E | short N | C | |
| 17663347 | ppa025296m | CYP81E | 6 | + | 255 | C | | C | |
| 17663349 | ppa022979m | CYP76G | 7 | − | 144 | C | | C | |
| 17663536 | ppa015174m | | 1 | + | 0 | F | 153aa | F | |
| 17663546 | ppa004768m | CYP90C | 7 | + | 51 | c | short N 2aa | C | |
| 17663708 | ppa004476m | CYP704A | 2 | + | 461 | C | | C | |
| 17663771 | ppa023168m | CYP707A | 8 | − | 1 | E | short N | C | |
| 17663967 | ppa023035m | | 2 | + | 0 | F | 194aa | F | |
| 17664051 | ppa016261m | CYP72A | 4 | + | 364 | E | many defects | U | |
| 17664163 | ppa027023m | CYP88A | 2 | + | 16 | C | | C | |
| 17664171 | ppa016800m | CYP734A | 1 | − | 374 | E | short N | C | |
| 17664453 | ppa023335m | CYP92A | 7 | + | 173 | C | | C | |
| 17664458 | | | 1 | + | 0 | F | 207aa | F | |
| 17664495 | ppa004152m | CYP71A | 5 | + | 119 | C | | C | |
| 17664572 | ppa017619m | CYP93B | 6 | − | 232 | C | | C | |
| 17664580 | ppa017339m | CYP71A | 5 | − | 119 | C | | C | |
| 17664597 | ppa025083m | CYP71A | 4 | + | 106 | E | extra I | c | FS |
| 17664651 | ppa021548m | | 6 | + | 0 | F | 209aa | F | |
| 17664725 | ppa015645m | | 1 | + | 0 | F | 181aa | F | |
| 17664728 | ppa004149m | CYP714A | 2 | + | 399 | C | | C | |
| 17664929 | ppa026707m | | 6 | + | 0 | F | 242aa | F | |
| 17664985 | ppa023213m | CYP76A | 7 | − | 142 | E | extra I x 2 | c | FS |
| 17665004 | ppa006307m | CYP72A | 4 | + | 351 | E | missing E | C | |
| 17665119 | ppa026605m | CYP79A | 7 | − | 313 | E | short N | C | |
| 17665137 | ppa026417m | | 1 | + | 0 | F | 92aa | F | |
| 17665290 | ppa004298m | | 1 | − | 453 | E | short N; extra C | C | |
| 17665457 | | | 6 | − | 0 | F | 68aa | F | |
| 17665472 | ppa005060m | CYP90A | 2 | + | 47 | C | | C | |
| 17665595 | ppa024077m | CYP71A | 4 | + | 106 | E | extra I | c | FS |
| 17665598 | ppa022174m | CYP82A | 1 | − | 211 | C | | C | |
| 17665760 | ppa022752m | CYP82A | 1 | − | 374 | E | short N | C | |
| 17665784 | ppa026638m | | 1 | + | 0 | F | 254aa | F | |
| 17665907 | ppa020783m | | 1 | − | 0 | F | 334aa | F | |
| 17665942 | ppa017987m | CYP81E | 6 | + | 255 | E | extra I; short C | C | |
| 17665951 | ppa020957m | CYP724A | 3 | − | 43 | C | | C | |
| 17666006 | ppa025192m | | 1 | − | 0 | F | 70a | F | |
| 17666016 | ppa026390m | | 4 | − | 0 | F | 373aa | F | |
| 17666021 | ppa025696m | CYP71D | 4 | − | 71 | E | short C | c | FS |
| 17666096 | ppa004301m | CYP82C | 8 | + | 223 | C | | C | |
| 17666272 | ppa017479m | CYP71D | 4 | − | 71 | E | many defects | C | |
| 17666294 | ppa021021m | | 1 | + | 0 | F | 239aa | F | |
| 17666318 | | AGE | 5 | − | 0 | X | 423aa | X | |
| 17666877 | ppa004901m | CYP88A | 2 | − | 16 | C | | C | |
| 17666896 | ppa019933m | CYP86C | 1 | + | 425 | C | | C | |
| 17666907 | ppa004517m | CYP99A | 1 | + | 149 | C | | C | |
| 17667063 | ppa019731m | CYP82C | 6 | − | 214 | E | short N | C | |
| 17667246 | ppa026871m | CYP78A | 1 | + | 272 | C | | C | |
| 17667258 | ppa026692m | CYP81E | 6 | + | 255 | E | extra I | C | |
| 17667471 | ppa022621m | CYP374A | 1 | + | 374 | c | 8aa short N | C | |
| 17667486 | ppa016799m | | 5 | − | 0 | F | 192aa | F | |
| 17667725 | ppa016144m | CYP98A | 5 | + | 167 | C | | C | |
| 17667852 | ppa025066m | CYP71D | 4 | − | 71 | C | | C | |
| 17668013 | ppa004984m | | 4 | + | 0 | U | 100N | U | |
| 17668213 | ppa004436m | CYP92A | 3 | + | 174 | C | | C | |
| 17668232 | ppa023786m | CYP81E | 6 | + | 255 | C | | C | |
| 17668327 | ppa019549m | | 5 | + | 0 | F | 289aa | F | |
| 17668446 | ppa004495m | CYP704A | 2 | − | 461 | C | | C | |
| 17668474 | ppa004433m | CYP75B | 5 | − | 161 | C | | C | |
| 17668526 | ppa002142m | reductase | 1 | + | 477 | X | | X | |
| 17668529 | ppa004463m | CYP72B1 | 1 | + | 374 | C | | C | |
| 17668564 | ppa004579m | CYP704A | 2 | + | 456 | C | | C | |
| 17668595 | ppa021318m | | 6 | + | 0 | F | 400aa | F | |
| 17668713 | ppa004473m | CYP98A | 1 | + | 163 | C | | C | |

| | | | | | | | | |
|---|---|---|---|---|---|---|---|---|
| 17668998 | ppa017753m | CYP718 | 3 | + | 42 | E | short N | C |
| 17669024 | ppa003868m | CYP78A | 8 | + | 269 | C | | C |
| 17669152 | ppa022041m | CYP92A | 6 | − | 188 | C | | C |
| 17669173 | ppa004377m | CYP71A | 5 | + | 186 | E | extra I | C |
| 17669299 | ppa003821m | CYP51 | 1 | + | 323 | E | extra N | C |
| 17669344 | ppa016810m | CYP703A | 2 | − | 320 | E | short N | C |

| | | Peach Phytozome | | Peach Refgs |
|---|---|---|---|---|
| Correct | C | 136 | C | 200 |
| Nearly good | c | 6 | c | 14 |
| Uncertain | U | 2 | U | 3 |
| Error | E | 78 | E | 5 |
| Pseudogene | P | 5 | P | 5 |
| Filtered | F | 73 | F | 73 |
| Non P450 | X | 3 | X | 3 |
| | Total | 303 | | 303 |

reductase    Cytochrome P450 reductase
AGE    l-Ala-D/L-Glu epimerase-like

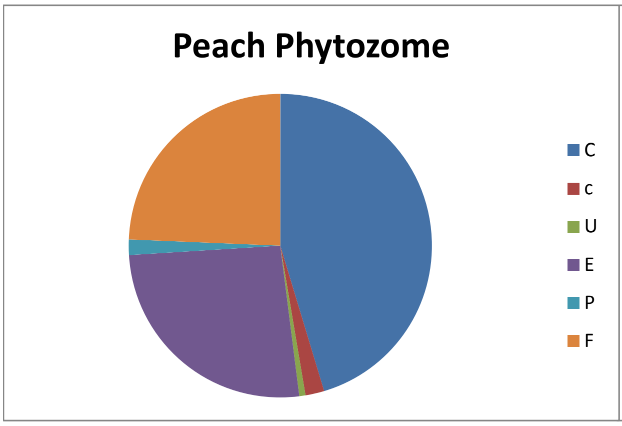


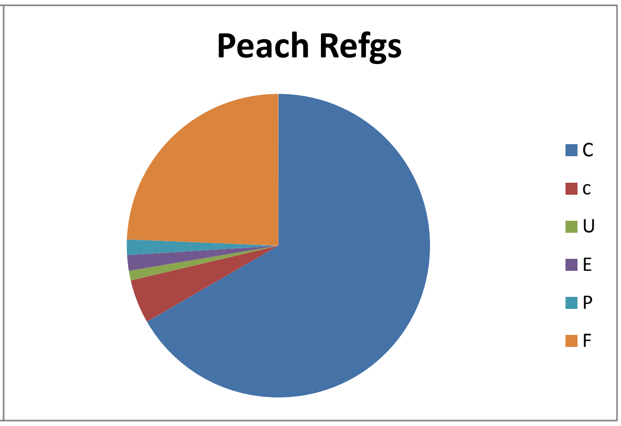

Table S2B Manual Assessment of Maize P450 Genes

| KOG-PID | Subfamily | Chrom | Strand | Cluster | Phytozome | Comment | Refgs | Comment |
|---|---|---|---|---|---|---|---|---|
| 20861891 | CYP709B | 1 | + | 391 | C | | C | |
| 20862038 | CYP071xn | 1 | + | 99 | P | short C | P | extra E |
| 20862133 | CYP078a | 1 | + | 279 | C | | C | |
| 20862210 | CYP081b | 1 | + | 267 | C | | C | |
| 20862211 | CYP081e | 1 | + | 0 | F | | F | |
| 20862212 | CYP081e | 1 | + | 0 | F | | F | |
| 20862316 | CYP076f | 1 | + | 0 | F | | F | |
| 20862635 | CYP092a | 1 | + | 180 | E | short C | c | FS |
| 20862842 | CYP97C | 1 | + | 406 | E | truncated | C | |
| 20862843 | CYP97C | 1 | + | 0 | F | | F | |
| 20863960 | CYP709B | 1 | + | 392 | C | | C | |
| 20864334 | CYP085a | 1 | + | 52 | C | | C | |
| 20864806 | CYP081b | 1 | + | 267 | C | | C | |
| 20864817 | CYP081b | 1 | + | 267 | C | | C | |
| 20865126 | CYP071j | 1 | + | 92 | C | | C | |
| 20865138 | CYP078a | 1 | + | 269 | C | | c | 2aa shor N |
| 20865139 | CYP078a | 1 | + | 0 | F | | F | |
| 20865679 | CYP87A | 1 | + | 0 | F | | F | |
| 20866187 | CYP709B | 1 | + | 392 | C | | C | |
| 20866772 | CYP706a | 1 | + | 197 | C | | C | |
| 20867226 | CYP071j | 1 | + | 90 | C | | C | |
| 20868683 | CYP084a | 1 | + | 185 | C | | C | |
| 20868809 | CYP20D | 1 | + | 436 | C | | C | |
| 20868976 | CYP099a | 1 | + | 191 | C | | C | |
| 20868977 | CYP099a | 1 | + | 0 | F | | F | |
| 20869162 | CYP86B | 1 | + | 424 | C | | C | |
| 20869439 | CYP092a | 1 | + | 180 | C | | C | |
| 20869610 | CYP081b | 1 | + | 267 | C | | C | |
| 20869665 | CYP94D | 1 | + | 451 | C | | C | |
| 20869793 | CYP081b | 1 | + | 267 | C | | C | |
| 20869898 | CYP074a | 1 | + | 467 | C | | C | |
| 20869899 | CYP074a | 1 | + | 0 | F | | F | |
| 20871007 | CYP071e | 1 | + | 132 | C | | C | |
| 20861868 | CYP86A | 1 | − | 0 | F | | F | |
| 20862296 | CYP074a | 1 | − | 467 | C | | C | |
| 20862565 | CYP076f | 1 | − | 172 | E | short C | c | FS |
| 20863233 | CYP081b | 1 | − | 268 | E | extra I | C | |
| 20863430 | CYP704B | 1 | − | 453 | C | | C | |
| 20863749 | CYP071x | 1 | − | 96 | C | | C | |
| 20863837 | CYP074a | 1 | − | 467 | C | | C | |
| 20864094 | CYP714A | 1 | − | 397 | C | | C | |
| 20864338 | CYP090b | 1 | − | 46 | C | | c | 4aa short N |
| 20864339 | CYP090b | 1 | − | 0 | F | | F | |
| 20864340 | CYP090b | 1 | − | 0 | F | | F | |
| 20864341 | CYP090b | 1 | − | 0 | F | | F | |
| 20865193 | CYP89A | 1 | − | 300 | c | long N | c | short N |
| 20865194 | CYP89A | 1 | − | 0 | F | | F | |
| 20865195 | CYP89A | 1 | − | 0 | F | | F | |
| 20865850 | CYP20D | 1 | − | 436 | C | | C | |
| 20866364 | CYP071x | 1 | − | 96 | C | | C | |
| 20866385 | CYP94D | 1 | − | 451 | C | | C | |
| 20866403 | CYP088a | 1 | − | 66 | C | | C | |
| 20866578 | CYP078a | 1 | − | 281 | C | | C | |
| 20866622 | CYP081b | 1 | − | 0 | F | | F | |
| 20867075 | CYP96A | 1 | − | 0 | F | | F | |
| 20867248 | CYP076f | 1 | − | 158 | C | | C | |
| 20867287 | CYP085a | 1 | − | 52 | P | too long | P | too short |
| 20867289 | CYP081b | 1 | − | 267 | C | | C | |
| 20867576 | CYP078a | 1 | − | 273 | C | | C | |
| 20867577 | CYP078a | 1 | − | 0 | F | | F | |
| 20867578 | CYP078a | 1 | − | 0 | F | | F | |
| 20867950 | CYP704A | 1 | − | 460 | C | | C | |
| 20868491 | CYP079a | 1 | − | 318 | C | | E | I1 position |
| 20869229 | CYP707A | 1 | − | 0 | F | | F | |
| 20869734 | CYP081b | 1 | − | 268 | C | | C | |
| 20869762 | CYP709B | 1 | − | 0 | F | | F | |
| 20869831 | CYP707a | 1 | − | 1 | C | | C | |
| 20869832 | CYP707a | 1 | − | 0 | F | | F | |
| 20871474 | CYP071xn | 1 | − | 92 | C | | C | |
| 20871476 | CYP89A | 1 | − | 0 | F | | F | |
| 20841659 | CYP714A | 2 | + | 397 | C | | C | |
| 20841660 | CYP714A | 2 | + | 0 | F | | F | |
| 20841661 | CYP714A | 2 | + | 0 | F | | F | |
| 20841662 | CYP714A | 2 | + | 0 | F | | F | |
| 20841738 | CYP727a | 2 | + | 469 | C | | C | |
| 20841754 | CYP092a | 2 | + | 175 | C | | C | |
| 20842664 | CYP071c | 2 | + | 102 | C | | C | |
| 20843216 | CYP051a | 2 | + | 327 | C | | C | |
| 20843217 | CYP051a | 2 | + | 0 | F | | F | |

| | | | | | | | | | |
|---|---|---|---|---|---|---|---|---|---|
| 20843487 | reductase | 2 | + | 475 | C | | C | |
| 20843488 | CYP102a | 2 | + | 0 | F | | F | |
| 20843759 | CYP092a | 2 | + | 182 | C | | E | short N, short C |
| 20843948 | CYP071a | 2 | + | 121 | C | | E | extra E |
| 20844027 | CYP071a | 2 | + | 98 | C | | C | |
| 20844765 | CYP090a | 2 | + | 48 | C | | C | |
| 20845128 | CYP071j | 2 | + | 88 | C | | c | 5aa short N |
| 20845355 | CYP725a | 2 | + | 22 | C | | C | |
| 20846847 | CYP071a | 2 | + | 98 | C | | C | |
| 20846848 | CYP071a | 2 | + | 0 | F | | F | |
| 20846998 | CYP86A | 2 | + | 422 | E | extra N | C | |
| 20846999 | CYP86A | 2 | + | 0 | F | | F | |
| 20847000 | CYP86A | 2 | + | 0 | F | | F | |
| 20847595 | CYP704A | 2 | + | 459 | C | | C | |
| 20848671 | CYP709B | 2 | + | 392 | C | | C | |
| 20842668 | CYP092a | 2 | − | 180 | c | long N | c | short N |
| 20842772 | CYP078a | 2 | − | 279 | C | | C | |
| 20843195 | CYP94B | 2 | − | 446 | c | long N | c | short N |
| 20843196 | CYP94B | 2 | − | 0 | F | | F | |
| 20843229 | CYP89A | 2 | − | 306 | C | | C | |
| 20843559 | CYP081b | 2 | − | 267 | C | | C | |
| 20843807 | CYP092a | 2 | − | 180 | U | short C | U | E2? |
| 20844064 | CYP092a | 2 | − | 0 | F | | F | |
| 20845006 | CYP89A | 2 | − | 0 | F | | F | |
| 20845470 | CYP89A | 2 | − | 0 | F | | F | |
| 20845572 | CYP098a | 2 | − | 0 | F | | F | |
| 20845605 | CYP724A | 2 | − | 43 | C | | c | 4aa short N |
| 20845632 | CYP709B | 2 | − | 392 | C | | C | |
| 20846126 | CYP092a | 2 | − | 180 | C | | C | |
| 20846478 | CYP071xn | 2 | − | 0 | F | | F | |
| 20846789 | CYP071xn | 2 | − | 99 | E | too long | C | |
| 20846790 | CYP071xn | 2 | − | 0 | F | | F | |
| 20847212 | CYP071xn | 2 | − | 99 | c | long N | c | short N |
| 20847225 | CYP707a | 2 | − | 12 | E | long E3 | C | |
| 20847632 | CYP77A | 2 | − | 285 | c | long N | c | short N |
| 20848411 | CYP071xn | 2 | − | 99 | C | | C | |
| 20849062 | CYP071xn | 2 | − | 94 | C | | C | |
| 20825119 | CYP89A | 3 | + | 0 | F | | F | |
| 20825320 | CYP89A | 3 | + | 0 | F | | F | |
| 20826380 | CYP706a | 3 | + | 197 | C | short N | C | |
| 20826447 | CYP098a | 3 | + | 164 | C | | C | |
| 20826448 | CYP098a | 3 | + | 0 | F | | F | |
| 20826449 | CYP098a | 3 | + | 0 | F | | F | |
| 20826450 | CYP098a | 3 | + | 0 | F | | F | |
| 20826451 | CYP098a | 3 | + | 0 | F | | F | |
| 20826839 | CYP89A | 3 | + | 305 | C | | C | |
| 20827143 | CYP005a | 3 | + | 331 | E | short N | C | |
| 20828808 | CYP734A | 3 | + | 383 | C | | C | |
| 20828809 | CYP734A | 3 | + | 0 | F | | F | |
| 20830181 | CYP072x | 3 | + | 357 | C | | C | |
| 20830182 | CYP072x | 3 | + | 0 | F | | F | |
| 20830183 | CYP072x | 3 | + | 0 | F | | F | |
| 20830970 | CYP094a | 3 | + | 452 | C | | C | |
| 20831245 | CYP051a | 3 | + | 323 | C | | C | |
| 20831246 | CYP051a | 3 | + | 0 | F | | F | |
| 20831357 | CYP072x | 3 | + | 0 | F | | F | |
| 20831429 | CYP003a | 3 | + | 0 | F | | F | |
| 20831431 | CYP710A | 3 | + | 470 | C | | E | short N |
| 20831571 | AGE | 3 | + | 473 | X | | X | |
| 20831572 | CYP513F | 3 | + | 0 | F | | F | |
| 20825778 | CYP051a | 3 | − | 327 | c | long N | c | short N |
| 20826398 | CYP709B | 3 | − | 393 | C | | C | |
| 20826581 | CYP072x | 3 | − | 357 | C | | C | |
| 20826582 | CYP072x | 3 | − | 0 | F | | F | |
| 20828298 | CYP072x | 3 | − | 0 | F | | F | |
| 20829114 | CYP072x | 3 | − | 360 | C | | C | |
| 20829155 | CYP071j | 3 | − | 90 | E | exon 1, short C | C | |
| 20829156 | CYP071j | 3 | − | 0 | F | | F | |
| 20829812 | CYP94C | 3 | − | 441 | C | | C | |
| 20830676 | CYP086a | 3 | − | 418 | C | | E | short C |
| 20831103 | CYP071a | 3 | − | 97 | C | | C | |
| 20831331 | CYP90D | 3 | − | 50 | C | | C | |
| 20831332 | CYP90D | 3 | − | 0 | F | | F | |
| 20831483 | CYP072x | 3 | − | 359 | C | | C | |
| 20831484 | CYP072x | 3 | − | 0 | F | | F | |
| 20831551 | CYP072x | 3 | − | 360 | C | | C | |
| 20831552 | CYP072x | 3 | − | 0 | F | | F | |
| 20855236 | CYP714A | 4 | + | 0 | F | | F | |
| 20856296 | CYP071j | 4 | + | 90 | C | | C | |
| 20856392 | CYP716A | 4 | + | 23 | C | | C | |
| 20856684 | CYP071j | 4 | + | 94 | C | | C | |
| 20857681 | CYP092a | 4 | + | 178 | c | long N | c | short N |

| | | | | | | | | |
|---|---|---|---|---|---|---|---|---|
| 20858029 | CYP89A | 4 | + | 307 | C | | C | |
| 20858857 | CYP051a | 4 | + | 324 | C | | C | |
| 20858993 | CYP071c | 4 | + | 101 | c | long N | c | short N |
| 20858994 | CYP071c | 4 | + | 0 | F | | F | |
| 20859035 | CYP005a | 4 | + | 332 | U | E3 | U | E3 |
| 20859036 | CYP6AG | 4 | + | 0 | F | | F | |
| 20859704 | CYP707a | 4 | + | 6 | C | | C | |
| 20859705 | CYP707a | 4 | + | 0 | F | | F | |
| 20859921 | CYP071c | 4 | + | 103 | C | | C | |
| 20859964 | CYP97C | 4 | + | 0 | F | | F | |
| 20860740 | reductase | 4 | + | 482 | X | | X | |
| 20860984 | CYP89A | 4 | + | 303 | C | | C | |
| 20861088 | reductase | 4 | + | 475 | C | | C | |
| 20861311 | CYP081b | 4 | + | 267 | c | long N | c | short N |
| 20854891 | CYP090a | 4 | − | 48 | C | | C | |
| 20854892 | CYP090a | 4 | − | 0 | F | | F | |
| 20854893 | CYP090a | 4 | − | 0 | F | | F | |
| 20854894 | CYP090a | 4 | − | 0 | F | | F | |
| 20855588 | CYP078a | 4 | − | 279 | C | | C | |
| 20855748 | CYP715A | 4 | − | 394 | C | | C | |
| 20856144 | CYP75B | 4 | − | 162 | E | many E | C | |
| 20856145 | CYP075b | 4 | − | 0 | F | | F | |
| 20856439 | CYP20D | 4 | − | 436 | C | | C | |
| 20857860 | CYP071c | 4 | − | 103 | C | | C | |
| 20858061 | CYP071c | 4 | − | 103 | E | extra E | C | |
| 20858062 | CYP071c | 4 | − | 0 | F | | F | |
| 20858063 | CYP071c | 4 | − | 0 | F | | F | |
| 20858064 | CYP071c | 4 | − | 0 | F | | F | |
| 20858065 | CYP071c | 4 | − | 0 | F | | F | |
| 20858123 | CYP074a | 4 | − | 467 | C | | C | |
| 20858525 | CYP071j | 4 | − | 88 | C | | C | |
| 20858526 | CYP071j | 4 | − | 0 | F | | F | |
| 20858877 | CYP86C | 4 | − | 425 | C | | E | extra E |
| 20858878 | CYP86C | 4 | − | 0 | F | | F | |
| 20858957 | CYP94B | 4 | − | 446 | C | | C | |
| 20859127 | CYP071a | 4 | − | 243 | C | | E | I1 position, extra intron |
| 20859586 | CYP071j | 4 | − | 94 | E | short N | C | |
| 20860022 | CYP099a | 4 | − | 191 | C | | C | |
| 20860249 | CYP099a | 4 | − | 191 | C | | C | |
| 20860251 | CYP075b | 4 | − | 162 | C | | C | |
| 20860448 | CYP89A | 4 | − | 307 | c | long N | c | short N |
| 20860477 | CYP707a | 4 | − | 14 | C | | E | extra I |
| 20860478 | CYP707a | 4 | − | 0 | F | | F | |
| 20860886 | CYP735A | 4 | − | 388 | E | missing E1 | C | |
| 20860938 | CYP097b | 4 | − | 412 | C | | C | |
| 20860939 | CYP097b | 4 | − | 0 | F | | F | |
| 20877138 | CYP071d | 5 | + | 93 | C | | C | |
| 20877773 | CYP071j | 5 | + | 88 | E | short C | c | FS |
| 20877774 | CYP071j | 5 | + | 0 | F | | F | |
| 20878227 | CYP734A | 5 | + | 383 | E | retained I | C | |
| 20878266 | CYP073a | 5 | + | 207 | c | long N | c | short N |
| 20878439 | CYP87A | 5 | + | 56 | C | | E | extra NE |
| 20879413 | CYP94B | 5 | + | 0 | F | | F | |
| 20879434 | CYP079a | 5 | + | 318 | C | | C | |
| 20880195 | CYP97C | 5 | + | 0 | F | | F | |
| 20880786 | CYP084a | 5 | + | 185 | C | | C | |
| 20880856 | CYP071j | 5 | + | 88 | C | | C | |
| 20881054 | CYP076f | 5 | + | 158 | C | | C | |
| 20881436 | CYP87A | 5 | + | 63 | C | | C | |
| 20881481 | CYP071j | 5 | + | 91 | C | | C | |
| 20881482 | CYP071j | 5 | + | 0 | F | | F | |
| 20881975 | CYP86B | 5 | + | 424 | C | | c | 4aa short N |
| 20882219 | CYP77B | 5 | + | 286 | C | | C | |
| 20882339 | CYP87A | 5 | + | 0 | F | | F | |
| 20882386 | CYP074a | 5 | + | 467 | C | | C | |
| 20876354 | CYP071d | 5 | − | 93 | C | | C | |
| 20876475 | CYP071j | 5 | − | 92 | c | long N | c | short N |
| 20877274 | CYP89A | 5 | − | 300 | C | | C | |
| 20877539 | CYP86A | 5 | − | 422 | C | | C | |
| 20878287 | CYP725a | 5 | − | 24 | C | | C | |
| 20878711 | CYP081b | 5 | − | 267 | C | | C | |
| 20878781 | CYP075b | 5 | − | 162 | C | | C | |
| 20879184 | CYP081b | 5 | − | 267 | C | | C | |
| 20879876 | CYP071j | 5 | − | 91 | E | short N | C | |
| 20879960 | CYP97A | 5 | − | 410 | C | | C | |
| 20881730 | CYP89A | 5 | − | 305 | E | short N | C | |
| 20881918 | CYP081b | 5 | − | 267 | C | | C | |
| 20882387 | CYP093a | 5 | − | 233 | E | short C | C | |
| 20882764 | CYP707a | 5 | − | 6 | C | | C | |
| 20882765 | CYP707a | 5 | − | 0 | F | | F | |
| 20883328 | CYP079a | 5 | − | 318 | C | | C | |
| 20820934 | CYP94C | 6 | + | 441 | C | | C | |

| | | | | | | | | |
|---|---|---|---|---|---|---|---|---|
| 20821377 | CYP071xn | 6 | + | 100 | C | | C | |
| 20821686 | CYP094a | 6 | + | 452 | C | | C | |
| 20821801 | CYP89A | 6 | + | 307 | C | | c | 6aa short N |
| 20822852 | CYP098a | 6 | + | 164 | C | | C | |
| 20823436 | CYP071xn | 6 | + | 100 | C | | E | extra last intron |
| 20823568 | CYP073a | 6 | + | 207 | C | | C | |
| 20824162 | CYP20D | 6 | + | 466 | C | | C | |
| 20824284 | CYP714A | 6 | + | 0 | F | | F | |
| 20820309 | CYP071j | 6 | − | 89 | C | | C | |
| 20820629 | CYP709B | 6 | − | 392 | C | | C | |
| 20820922 | CYP004a | 6 | − | 0 | F | | F | |
| 20822302 | CYP704A | 6 | − | 464 | C | | C | |
| 20822813 | CYP90D | 6 | − | 50 | C | | C | |
| 20823056 | CYP071j | 6 | − | 88 | C | | C | |
| 20823861 | CYP709B | 6 | − | 392 | C | | E | extra last intron |
| 20832176 | CYP071a | 7 | + | 98 | C | | C | |
| 20832510 | CYP709B | 7 | + | 0 | F | | F | |
| 20832598 | CYP709B | 7 | + | 392 | C | | C | |
| 20833544 | reductase | 7 | + | 475 | C | | C | |
| 20834344 | CYP078a | 7 | + | 273 | C | | C | |
| 20835393 | CYP97A | 7 | + | 0 | F | | F | |
| 20836509 | CYP092a | 7 | + | 175 | C | | C | |
| 20831976 | CYP734A | 7 | − | 384 | C | | C | |
| 20832042 | CYP725a | 7 | − | 22 | C | | C | |
| 20833130 | CYP707a | 7 | − | 12 | C | | C | |
| 20833131 | CYP707a | 7 | − | 0 | F | | F | |
| 20833132 | CYP707a | 7 | − | 0 | F | | F | |
| 20833532 | CYP735A | 7 | − | 388 | C | | C | |
| 20833533 | CYP735A | 7 | − | 0 | F | | F | |
| 20833602 | CYP076f | 7 | − | 170 | C | | C | |
| 20834139 | CYP092a | 7 | − | 182 | C | | C | |
| 20834884 | CYP081b | 7 | − | 267 | C | | C | |
| 20835484 | CYP076f | 7 | − | 172 | E | lack E1 | c | N end |
| 20849347 | CYP071x | 8 | + | 0 | F | | F | |
| 20849732 | CYP704A | 8 | + | 459 | E | short N | C | |
| 20850107 | CYP071a | 8 | + | 97 | C | | C | |
| 20850684 | CYP071a | 8 | + | 97 | C | | C | |
| 20850910 | CYP071a | 8 | + | 97 | C | | C | |
| 20851408 | CYP072x | 8 | + | 0 | F | | F | |
| 20851488 | CYP082a | 8 | + | 346 | C | | C | |
| 20851541 | CYP96A | 8 | + | 0 | F | | F | |
| 20851717 | CYP071a | 8 | + | 0 | F | | F | |
| 20851920 | CYP94C | 8 | + | 441 | C | | C | |
| 20852050 | CYP071a | 8 | + | 97 | C | | C | |
| 20852951 | CYP072x | 8 | + | 359 | C | | C | |
| 20853056 | CYP722A | 8 | + | 2 | U | | U | |
| 20853057 | CYP722A | 8 | + | 0 | F | | F | |
| 20853070 | CYP072x | 8 | + | 358 | C | | C | |
| 20853283 | CYP073a | 8 | + | 207 | C | | C | |
| 20853311 | CYP20D | 8 | + | 436 | C | | C | |
| 20853312 | CYP96A | 8 | + | 0 | F | | F | |
| 20853386 | CYP86B | 8 | + | 0 | F | | F | |
| 20853732 | CYP072x | 8 | + | 358 | C | | C | |
| 20853746 | CYP075b | 8 | + | 162 | C | | C | |
| 20849226 | CYP081b | 8 | − | 267 | C | | C | |
| 20849227 | CYP081b | 8 | − | 0 | F | | F | |
| 20849588 | CYP86C | 8 | − | 0 | F | | F | |
| 20849901 | CYP709B | 8 | − | 392 | C | | C | |
| 20850447 | AGE | 8 | − | 473 | X | | X | |
| 20850987 | CYP051a | 8 | − | 327 | C | | C | |
| 20851000 | CYP89A | 8 | − | 303 | C | | C | |
| 20851164 | CYP072x | 8 | − | 357 | E | extra E | C | |
| 20851165 | CYP072x | 8 | − | 0 | F | | F | |
| 20851166 | CYP072x | 8 | − | 0 | F | | F | |
| 20851811 | CYP073a | 8 | − | 207 | C | | C | |
| 20851954 | CYP072x | 8 | − | 359 | C | | C | |
| 20852021 | CYP086a | 8 | − | 418 | C | | E | short C |
| 20836969 | CYP20D | 9 | + | 436 | C | | C | |
| 20837064 | CYP088a | 9 | + | 17 | C | | c | 5aa short N |
| 20837272 | CYP093a | 9 | + | 233 | C | | C | |
| 20837471 | CYP704A | 9 | + | 0 | F | | F | |
| 20837538 | CYP071x | 9 | + | 0 | F | | F | |
| 20839040 | CYP075a | 9 | + | 0 | F | | F | |
| 20839283 | CYP734A | 9 | + | 383 | C | | C | |
| 20840262 | CYP704A | 9 | + | 0 | F | | F | |
| 20840282 | CYP088a | 9 | + | 17 | C | | C | |
| 20840639 | CYP075a | 9 | + | 0 | F | | F | |
| 20840694 | CYP078a | 9 | + | 0 | F | | F | |
| 20841029 | CYP099a | 9 | + | 0 | F | | F | |
| 20837150 | CYP071j | 9 | − | 89 | C | | C | |
| 20837265 | CYP701a | 9 | − | 312 | C | | C | |
| 20837344 | CYP709B | 9 | − | 392 | C | | E | E1-4 |

| | | | | | | | | |
|---|---|---|---|---|---|---|---|---|
| 20837345 | CYP709B | 9 | – | 0 | F | | F | |
| 20837734 | CYP701a | 9 | – | 312 | C | | C | |
| 20837735 | CYP701a | 9 | – | 0 | F | | F | |
| 20837966 | CYP071x | 9 | – | 96 | C | | E | I1 3aa shift |
| 20838041 | CYP709B | 9 | – | 391 | C | | C | |
| 20838065 | CYP5A | 9 | – | 330 | C | | E | short N |
| 20838066 | CYP005a | 9 | – | 0 | F | | F | |
| 20838272 | CYP078a | 9 | – | 273 | U | | U | |
| 20838345 | CYP709B | 9 | – | 0 | F | | F | |
| 20838611 | CYP074a | 9 | – | 467 | C | | C | |
| 20838695 | CYP079a | 9 | – | 316 | C | | C | |
| 20838913 | CYP94D | 9 | – | 451 | C | | C | |
| 20839039 | CYP072x | 9 | – | 0 | F | | F | |
| 20839201 | CYP734A | 9 | – | 383 | C | | C | |
| 20839485 | CYP093a | 9 | – | 230 | C | | C | |
| 20841282 | CYP071x | 9 | – | 96 | C | | C | |
| 20871964 | CYP093a | 10 | + | 233 | C | | C | |
| 20872325 | CYP89A | 10 | + | 0 | F | | F | |
| 20872448 | CYP94C | 10 | + | 441 | C | | C | |
| 20872840 | reductase | 10 | + | 475 | X | | X | |
| 20873564 | CYP87A | 10 | + | 56 | C | | C | |
| 20875139 | reductase | 10 | + | 475 | X | | X | |
| 20875140 | reductase | 10 | + | 0 | F | | X | |
| 20875319 | CYP071c | 10 | + | 100 | E | missing E | C | |
| 20871820 | CYP704A | 10 | – | 459 | E | retained I | C | |
| 20871821 | CYP704A | 10 | – | 0 | F | | F | |
| 20872629 | CYP085a | 10 | – | 0 | F | | F | |
| 20872630 | CYP085a | 10 | – | 0 | F | | F | |
| 20872654 | CYP89A | 10 | – | 306 | C | | C | |
| 20872699 | CYP071a | 10 | – | 243 | E | eij shift | C | |
| 20872934 | CYP703a | 10 | – | 320 | C | | C | |
| 20872935 | CYP703a | 10 | – | 0 | F | | F | |
| 20873412 | CYP20D | 10 | – | 436 | C | | C | |
| 20873414 | CYP89A | 10 | – | 306 | C | | C | |
| 20873658 | CYP715A | 10 | – | 0 | F | | F | |
| 20873762 | CYP092a | 10 | – | 0 | F | | F | |
| 20873927 | CYP87A | 10 | – | 56 | C | | C | |
| 20874203 | CYP071c | 10 | – | 100 | C | | C | |
| 20874476 | CYP94C | 10 | – | 441 | C | | C | |
| 20874831 | CYP051a | 10 | – | 323 | c | long N | c | short N |
| 20874832 | CYP051a | 10 | – | 0 | F | | F | |
| 20875179 | CYP099a | 10 | – | 191 | C | | C | |
| 20875371 | CYP093a | 10 | – | 233 | E | missing E | C | |

| | | Maize Phytozome | | Maize Refgs |
|---|---|---|---|---|
| Correct | C | 201 | Correct | 200 |
| Nearly good | c | 13 | Nearly goc | 24 |
| Uncertain | U | 4 | Uncertain | 4 |
| Error | E | 25 | Error | 15 |
| Pseudogene | P | 2 | Pseudoger | 2 |
| Filtered | F | 118 | Filtered | 118 |
| Non P450 | X | 5 | Non P450 | 5 |
| | Total | 368 | | 368 |

reductase Cytochrome P450 reductase
AGE l-Ala-D/L-Glu epimerase-like

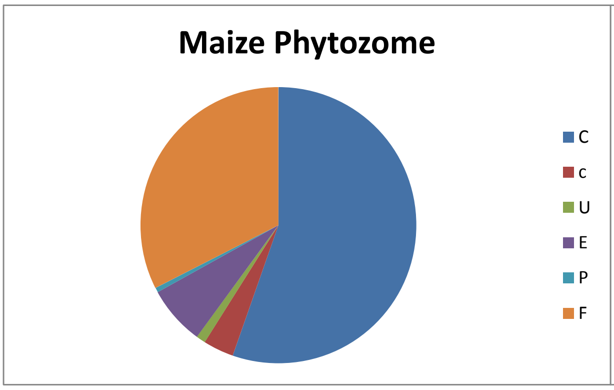


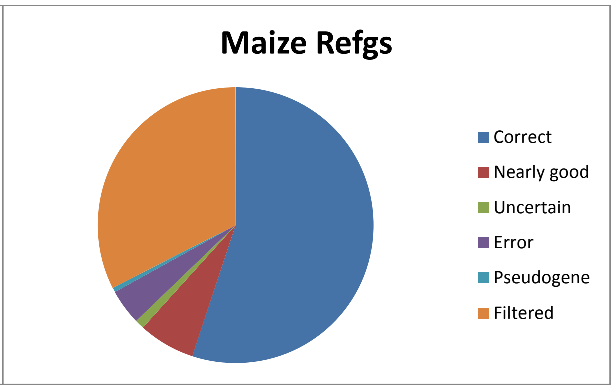

Table S3. Resources of Sequences and Annotations.

| SpecCode | Species | Genome/Aa/GFF | EST/cDNA |
|---|---|---|---|
| aquicoer | *Aquilegia coerulea* | Phytozome | PlantGDB, UniGene |
| arablyra | *Arabidopsis lyrata** | Phytozome | JGI |
| arabthal | *Arabidopsis thaliana* | Phytozome | PlantGDB, UniGene |
| astespec | *Asterochloris spec_Cgr/DA1pho* | JGI | JGI |
| bracdist | *Brachypodium distachyon** | Phytozome | PlantGDB, UniGene |
| brasrapa | *Brassica rapa* | Phytozome | PlantGDB |
| camesine | *Camellia sinensis* | Phytozome | PlantGDB, GenBank |
| capsrube | *Capsella rubella* | Phytozome | PlantGDB |
| caripapa | *Carica papaya** | Phytozome | PlantGDB, GenBank |
| chlarein | *Chlamydomonas reinhardtii** | Phytozome | PlantGDB, UniGene |
| chlovari | *Chlorella variabilis*_NC64A | JGI | JGI |
| chlovulg | *Chlorella vulgaris* | JGI | JGI |
| citrclem | *Citrus clementina* | Phytozome | PlantGDB, UniGene |
| coccsube | *Coccomyxa subellipsoidea* | Phytozome | PlantGDB |
| cucusati | *Cucumis sativus* | Phytozome | PlantGDB, GenBank |
| eucagran | *Eucalyptus grandis* | Phytozome | PlantGDB, GenBank |
| fragvesc | *Fragaria vesca* | Phytozome | PlantGDB, GenBank |
| glycmax_ | *Glycine max** | Phytozome | PlantGDB, UniGene |
| gossraim | *Gossypium raimondii* | Phytozome | PlantGDB, UniGene |
| jatrcurc | *Jatropha curcas* | Kazusa | PlantGDB, GenBank |
| linuusit | *Linum usitatissimum* | Phytozome | - |
| lotujapo | *Lotus japonicus* | Kazusa | PlantGDB, UniGene |
| maniescu | *Manihot esculenta* | Phytozome | PlantGDB, UniGene |
| meditrun | *Medicago truncatula** | Phytozome | PlantGDB, UniGene |
| micrpusi | *Micromonas pusilla** | Phytozome | JGI |
| micrrcc2 | *Micromonas* rcc299* | Phytozome | JGI, GenBank |
| mimugutt | *Mimulus guttatus* | Phytozome | PlantGDB, UniGene |
| oryzsati | *Oryza sativa** | Phytozome | PlantGDB, UniGene |
| ostrluci | *Ostreococcus lucimarinus** | Phytozome | PlantGDB, GenBank |
| ostrrcc8 | *Ostreococcus* rcc809 | JGI | JGI |
| ostrtaur | *Ostreococcus tauri** | JGI | JGI |
| panivirg | *Panicum virgatum* | Phytozome | - |

| | | | |
|---|---|---|---|
| phasvulg | *Phaseolus vulgaris* | Phytozome | PlantGDB, UniGene |
| physpate | *Physcomitrella patens** | Phytozome | PlantGDB, UniGene |
| poputric | *Populus trichocarpa** | Phytozome | PlantGDB, UniGene |
| prunpers | *Prunus persica* | Phytozome | PlantGDB, UniGene |
| ricicomm | *Ricinus communis** | Phytozome | PlantGDB, UniGene |
| selamoel | *Selaginella moellendorffii** | Phytozome | PlantGDB, UniGene |
| setaital | *Setaria italica* | Phytozome | - |
| solalyco | *Solanum lycopersicum** | Phytozome | PlantGDB, UniGene |
| solatube | *Solanum tuberosum** | Phytozome | PlantGDB, UniGene |
| sorgbico | *Sorghum bicolor* | Phytozome | PlantGDB, UniGene |
| thelhalo | *Thellungiella halophila* | Phytozome | PlantGDB, GenBank |
| theocaca | *Theobroma cacao* | Phytozome | PlantGDB, UniGene |
| vitivini | *Vitis vinifera** | Phytozome | PlantGDB, UniGene |
| volvcart | *Volvox carteri** | Phytozome | PlantGDB, UniGene |
| zea_mays | *Zea mays* | Phytozome | PlantGDB, UniGene |

| Resource | URL |
|---|---|
| Phytozome | http://www.phytozome.net/ |
| JGI | http://genome.jgi.doe.gov/ |
| Kazusa | http://www.kazusa.or.jp/e/index.html |
| PlantGDB | http://www.plantgdb.org/ |
| UniGene | http://www.ncbi.nlm.nih.gov/unigene |
| Genbank | http://www.ncbi.nlm.nih.gov/genbank/ |
